# Causal relationship between kidney stones and gut microbiota contributes to the gut-kidney axis: a two-sample Mendelian randomization study

**DOI:** 10.3389/fmicb.2023.1204311

**Published:** 2023-07-12

**Authors:** Minghui Liu, Youjie Zhang, Jian Wu, Meng Gao, Zewu Zhu, Hequn Chen

**Affiliations:** ^1^Department of Urology, Xiangya Hospital, Central South University, Changsha, China; ^2^National Clinical Research Center for Geriatric Disorders, Xiangya Hospital, Central South University, Changsha, China; ^3^Department of Internal Medicine, Section Endocrinology, Yale University School of Medicine, New Haven, CT, United States

**Keywords:** kidney stones, gut microbiota, *Oxalobacter formigenes*, Mendelian randomization, genus Subdoligranulum

## Abstract

**Background:**

Gut microbiota, particularly *Oxalobacter formigenes*, has been previously reported to be associated with kidney stones. However, the conflicting results from both observational and intervention studies have created substantial uncertainty regarding the contribution of *Oxalobacter formigenes* to the formation of kidney stone.

**Methods:**

We employed a two-sample MR analysis to investigate the causal relationship between gut microbiota and kidney stones using GWASs summary statistics obtained from the MiBioGen and FinnGen consortia. Moreover, we conducted a reserve MR analysis to assess the direction of the causal associations between gut microbiota and kidney stones. The inverse variance weighted (IVW) approach represents the primary method of Mendelian Randomization (MR) analysis.

**Results:**

Our analyses do not yield supportive evidence for a causal link between the genus *Oxalobacte*r (OR = 0.99, 95% CI: 0.90–1.09, *p* = 0.811) and the formation of kidney stones. The order *Actinomycetales* (OR = 0.79, 95% CI: 0.65–0.96, *p* = 0.020), family *Actinomycetaceae* (OR = 0.79, 95% CI: 0.65–0.96, *p* = 0.019), family *Clostridiaceae 1* (OR = 0.80, 95% CI: 0.67–0.96, *p* = 0.015), genus *Clostridiumsensustricto 1* (OR = 0.81, 95% CI: 0.67–0.98, *p* = 0.030) and genus *Hungatella* (OR = 0.86, 95% CI: 0.74–0.99, *p* = 0.040) had protective effects on kidney stones, and the genus *Haemophilus* (OR = 1.16, 95% CI: 1.01–1.33, *p* = 0.032), genus *Ruminococcaceae (UCG010)* (OR = 1.38, 95% CI: 1.04–1.84, *p* = 0.028), genus *Subdoligranulum* (OR = 1.27, 95% CI: 1.06–1.52, *p* = 0.009) were risk factors for kidney stones. Differential abundance analysis provide no evidence of a association between *Oxalobacter formigenes* and kidney stones, and showed genus Subdoligranulum were risk factors for kidney stones. Reverse MR analysis did not indicate any causal association of kidney stones on gut microbiota. No considerable heterogeneity of instrumental variables or horizontal pleiotropy was observed.

**Conclusion:**

Our two-sample MR study did not find any causal relationship between genus *Oxalobacter* and kidney stones. The association between gut microbiota and kidney stones does not solely depend on the presence of genus *Oxalobacter*/*Oxalobacter formigenes*. A more integrated approach using multiple omics platforms is needed to better understand the pathogenesis of kidney stones in the context of complex gene–environment interactions over time.

## Introduction

Kidney stones are a common chronic disease with a prevalence of 5–10%, and the rates appear to be rising in almost every country (Singh et al., 2022). Furthermore, the 10-year recurrence rate of kidney stones is 50% (Pearle et al., 2014), causing a significant burden on healthcare systems and having a substantial impact on patients’ quality of life. The etiology of kidney stones is highly complex and multifactorial, encompassing a range of factors including genetics, ethnicity, environment, dietary habits and metabolism. Despite significant research efforts, the precise mechanisms underlying the formation of kidney stone remain poorly understood. Thus, there is an urgent need to gain deeper insights into the pathogenesis of kidney stones and identify novel therapeutic targets that can be leveraged to develop more efficacious treatment strategies for this condition.

Gut microbiota, the complex community of microorganisms residing in the gastrointestinal tract, plays a crucial role in shaping the development and maintenance of the host’s health status through various interactions with the host, such as nutrient metabolism and immune regulation. Kidney stone is significantly influenced by various metabolic diseases, including hypertension, diabetes, and obesity, which have been found to be associated with alterations in the gut microbiota. In hypertensive patients, there is a significant reduction in microbial richness, diversity, and evenness, accompanied by a notable increase in the *Firmicutes/Bacteroidetes* ratio (Yang et al., 2015; Li et al., 2017). A study conducted on Chinese adolescents revealed a significant decrease in the abundance of *Bacteroides thetaiotaomicron* in obese individuals (Liu R. et al., 2017). A cohort study conducted in Sweden discovered a substantial reduction in gut microbial diversity among patients with type 2 diabetes mellitus (T2DM) (Wu et al., 2020). Recent researches have provided compelling evidence supporting a link between kidney stones and the gut microbiota (Mehta et al., 2016; Ticinesi et al., 2018; Stanford et al., 2020; Bostanghadiri et al., 2021). Studies have uncovered significant differences in the abundance of gut microbiota between kidney stone patients and healthy individuals (Ticinesi et al., 2018; Yuan et al., 2022). In fact, current researches on the relationship between kidney stones and gut microbiota have primarily focused on *Oxalobacter formigenes*, a bacterium that metabolizes oxalate as its principal energy source. This bacterium has been isolated from the fecal samples of individuals who have never experienced kidney stones, and its presence has been correlated with a reduction in urinary oxalate excretion (Siener et al., 2013). However, clinical studies have yielded inconsistent findings. *Oxalobacter formigenes* have been detected also in the fecal samples of individuals with a history of recurrent kidney stone formation. Interventions involving administration of *Oxalobacter formigenes* or other probiotic strains with oxalate-degrading capabilities have not consistently demonstrated a significant reduction in urinary oxalate excretion (Siva et al., 2009).

Therefore, further investigation is necessary to determine the specific contribution of *Oxalobacter formigenes* and other gut microbiota taxa to kidney stones. Mendelian randomization (MR) studies, akin to randomized controlled trials (RCTs), is an epidemiological approach for exploring the causal association between environmental exposures and diseases (Smith and Ebrahim, 2003; Swanson et al., 2017). Two-sample MR analysis allows for the integration of single-nucleotide polymorphisms (SNPs) data on exposure and outcome variables from independent genome-wide association studies (GWASs) to generate a unified causal estimate. SNPs adhere to the fundamental principle of random allocation of genetic variants during meiosis, which circumvents the influence of confounding factors and mitigates the risk of reverse causation, given that genetic variants precede the onset of the disease (Lawlor et al., 2008). This study employed a two-sample MR analysis to investigate the causal relationship between gut microbiota and kidney stones using GWASs summary statistics obtained from the MiBioGen and FinnGen consortia.

## Methods

### Exposure data

The instrumental variables utilized in our study were SNPs that were strongly associated with the composition of the human gut microbiome. These SNPs were selected from the largest GWASs dataset from the international consortium MiBioGen, involving 18,340 samples of 16S rRNA gene sequencing data from 24 population-based cohorts of various ancestries (Kurilshikov et al., 2021). A total of 211 gut microbiome taxa were included in the analysis, including 131 genera, 35 families, 20 orders, 16 classes, and 9 phyla.

### Outcome data

GWAS summary statistics for kidney stones were available from the 7th release of the FinnGen consortium, which consisted of 7,433 cases and 301,094 controls with the adjustment for age, sex, 10 principal components and genotyping batch. The cases were identified by the diagnosis codes of N20 in the *International Classification of Diseases, 10th Revision* (ICD-10) and 592 in ICD-8 and ICD-9.

### Instrumental variable selection

The three assumptions of MR are as follows: (1) Genetic variants have a strong and reliable association with the risk factors being studied; (2) Genetic variants are not associated with any confounding factors that could influence both the risk factors and the outcome; (3) Genetic variants only affect the outcome through their impact on the risk factors being investigated. SNPs associated with each gut microbiome were screened using the genome-wide significance threshold (*p* < 1 × 10^−5^) by referring to current MR studies on gut microbiome (Sanna et al., 2019). To ensure statistical independence, a linkage disequilibrium (LD) analysis (*R*^2^ < 0.001, clumping distance = 10,000 kb) was executed based on the European-based 1,000 Genome Projects. Furthermore, to ensure that the effects of the SNPs on each gut microbiota taxon correspond to the same allele as the effects on kidney stones, palindromic SNPs were removed. The *F*-statistic of instrumental variables was calculated to evaluate the extent of weak instrumental bias (F = beta^2^/se^2^). A *F*-statistic >10 was deemed as indicative of no bias caused by weak instrumental variables. To mitigate the correlation between SNPs and confounding factors, we utilized PhenoScanner^1^ to examine all incorporated instrumental variables, and subsequently removed SNPs that were associated with confounding factors.

### Statistical analysis

We employed five commonly used MR methods to investigate whether there was a causal relationship between gut microbiota and kidney stones, namely, the inverse-variance weighted (IVW), MR-Egger regression, weighted mode, weighted median and simple mode (Burgess et al., 2013; Bowden et al., 2015, 2016; Hartwig et al., 2017). IVW method was employed as the primary MR method to infer causality. IVW method assumes that all instrumental variables have a common causal effect on the outcome through the exposure. It combines the effect estimates of each instrumental variable in a meta-analysis-like framework, using the inverse of the variance of each effect estimate as a weight, to obtain a summary causal estimate. MR-Egger regression is a method used when there is horizontal pleiotropy, which occurs when the instrumental variables affect the outcome through pathways that are not mediated by the exposure. The MR-PRESSO analysis was also used to detect the presence of horizontal pleiotropy. After removing these outliers, the MR-PRESSO method re-estimates the causal effect using the remaining instrumental variables. By identifying and adjusting for horizontal pleiotropy in this way, the MR-PRESSO analysis can provide a more accurate and robust estimate of the causal effect in Mendelian randomization studies. Moreover, we conducted a reserve MR analysis to assess the direction of the causal associations between gut microbiota and kidney stones. Cochrane’s Q test was computed to evaluate the degree of heterogeneity observed among the effect estimates of SNPs. The scatter plots and funnel plots were created for visualizing the results of MR analyses and identifying potential outliers. “Leave-one-out” analysis was performed to identify potential heterogeneous SNPs. This approach involves sequentially removing one SNP at a time from the instrumental variable set and re-estimating the causal effect estimate. All statistical analyses in this study were conducted using the R software, version 4.1.3. The “TwoSampleMR” R package and the “MRPRESSO” R package were utilized in our MR study.

### Differential abundance analysis

We downloaded the raw data using the sequence read archive (SRA) accession numbers SRP140641, SRP140933, SRP066940, SRP103884, SRP125171 at https://github.com/amill017/USD_metaanalysis_2020 (Kachroo et al., 2021). The raw sequencing files of each sample were subjected to quality control using the fastp software. The quality-controlled sequencing data were processed using the Vsearch software to perform deduplication, denoising, and removal of chimeric sequences, resulting in a non-redundant Operational Taxonomic Units (OTU) table. The generated OTU table was imported into Qiime2, and species annotation was performed using available tools in Qiime2. Differential species analysis was conducted using the DESeq2 and Qiime2 packages in R software.

## Results

### SNPs selection

We obtained 102, 178, 215, 375, and 1,381 SNPs (*p* < 1 × 10^−5^) at the phylum, class, order, family, and genus levels, respectively. Namely, 2,251 SNPs were selected as instrumental variables. All F-statistics of instrumental variables were greater than 10.

### MR analyses

**Figure 1:**
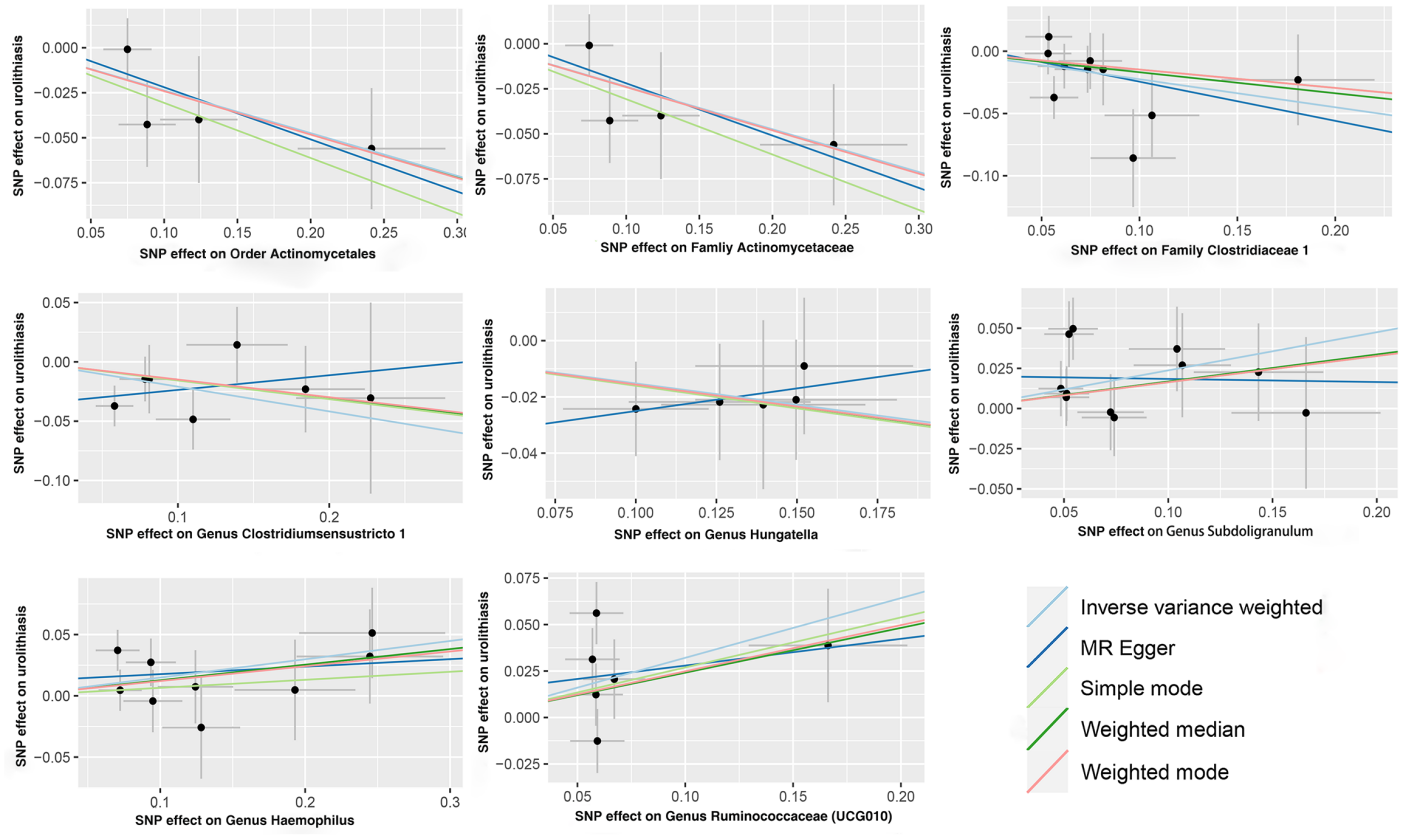
Scatter plots for the causal links between gut microbiota and kidney stones.

IVW analysis showed that the order Actinomycetales, family Actinomycetaceae, family Clostridiaceae 1, genus Clostridiumsensustricto 1, genus Haemophilus, genus Hungatella, genus Ruminococcaceae (UCG010), genus Subdoligranulum were associated with kidney stones, while there is no causal relationship between genus Oxalobacter (OR = 0.99, 95% CI: 0.90–1.09, *p* = 0.811) and kidney stones. The order Actinomycetales (OR = 0.79, 95% CI: 0.65–0.96, *p* = 0.020), family Actinomycetaceae (OR = 0.79, 95% CI: 0.65–0.96, *p* = 0.019), family Clostridiaceae 1 (OR = 0.80, 95% CI: 0.67–0.96, *p* = 0.015), genus Clostridiumsensustricto 1 (OR = 0.81, 95% CI: 0.67–0.98, *p* = 0.030) and genus Hungatella (OR = 0.86, 95% CI: 0.74–0.99, *p* = 0.040) had protective effects on kidney stones, and the genus Haemophilus (OR = 1.16, 95% CI: 1.01–1.33, *p* = 0.032), genus Ruminococcaceae (UCG010) (OR = 1.38, 95% CI: 1.04–1.84, *p* = 0.028), genus Subdoligranulum (OR = 1.27, 95% CI: 1.06–1.52, *p* = 0.009) were risk factors for kidney stones (Table 1). The scatter plots for the causal relationship between gut microbiota and kidney stones was presented in Figure 1. The specific instrumental variables used in MR analysis were listed in Supplementary Table S1. Supplementary Table S2 compiled the comprehensive results of all MR analyses conducted. Reverse MR analysis did not indicate any causal association of kidney stones on gut microbiota (Supplementary Table S3).

**Table 1:** MR results of causal links between gut microbiome and kidney stones risk.

Group	Gut microbiota	MR method	No.SNP	OR (95% CI)	*p*-value	*F*-statistic
Genus	Oxalobacter	Inverse variance weighted	11	0.99 (0.90–1.09)	0.811	21.35	MR Egger	11	0.78 (0.50–1.24)	0.319		Weighted median	11	0.97 (0.85–1.11)	0.667		Simple mode	11	0.96 (0.76–1.21)	0.729		Weighted mode	11	0.96 (0.77–1.20)	0.730	
Order	Actinomycetales	Inverse variance weighted	4	0.79 (0.65–0.96)	0.020	21.51	MR Egger	4	0.75 (0.48–1.16)	0.322		Weighted median	4	0.79 (0.61–1.02)	0.066		Simple mode	4	0.74 (0.52–1.04)	0.178		Weighted mode	4	0.79 (0.60–1.03)	0.183	
Family	Actinomycetaceae	Inverse variance weighted	4	0.79 (0.65–0.96)	0.019	21.57	MR Egger	4	0.75 (0.48–1.15)	0.320		Weighted median	4	0.79 (0.61–1.01)	0.058		Simple mode	4	0.74 (0.52–1.05)	0.188		Weighted mode	4	0.79 (0.60–1.03)	0.180	
Family	Clostridiaceae1	Inverse variance weighted	10	0.80 (0.67–0.96)	0.015	20.28	MR Egger	10	0.73 (0.43–1.24)	0.276		Weighted median	10	0.84 (0.66–1.08)	0.184		Simple mode	10	0.86 (0.61–1.22)	0.430		Weighted mode	10	0.86 (0.63–1.18)	0.386	
Genus	Clostridiumsensustricto 1	Inverse variance weighted	7	0.81 (0.67–0.98)	0.030	20.60	MR Egger	7	1.13 (0.70–1.83)	0.637		Weighted median	7	0.86 (0.66–1.12)	0.256		Simple mode	7	0.85 (0.58–1.26)	0.455		Weighted mode	7	0.86 (0.62–1.20)	0.417	
Genus	Haemophilus	Inverse variance weighted	9	1.16 (1.01–1.33)	0.032	23.42	MR Egger	9	1.06 (0.78–1.44)	0.705		Weighted median	9	1.14 (0.95–1.36)	0.172		Simple mode	9	1.07 (0.83–1.37)	0.626		Weighted mode	9	1.13 (0.89–1.42)	0.338	
Genus	Hungatella	Inverse variance weighted	5	0.86 (0.74–0.99)	0.040	20.43	MR Egger	5	1.17 (0.49–2.83)	0.744		Weighted median	5	0.85 (0.71–1.03)	0.098		Simple mode	5	0.85 (0.68–1.07)	0.243		Weighted mode	5	0.86 (0.68–1.07)	0.242	
Genus	Ruminococcaceae (UCG010)	Inverse variance weighted	6	1.38 (1.04–1.84)	0.028	21.43	MR Egger	6	1.15 (0.49–2.72)	0.759		Weighted median	6	1.27 (0.94–1.72)	0.115		Simple mode	6	1.31 (0.85–2.02)	0.280		Weighted mode	6	1.28 (0.92–1.79)	0.203	
Genus	Subdoligranulum	Inverse variance weighted	11	1.27 (1.06–1.52)	0.009	21.12	MR Egger	11	0.98 (0.62–1.55)	0.936		Weighted median	11	1.18 (0.93–1.51)	0.179		Simple mode	11	1.18 (0.85–1.63)	0.348		Weighted mode	11	1.18 (0.87–1.60)	0.317	

### Sensitivity analyses

According to the results of Cochran’s Q test, there was no statistically significant heterogeneity observed among these instrumental variables (Supplementary Table S4). MR-Egger regression intercept analysis showed that there was no directional horizontal pleiotropy for gut microbiota in kidney stones (Supplementary Table S5). Visual examination of scatter plots (Supplementary Figure S1) and leave-one-out plots (Supplementary Figure S2) revealed the possible existence of outliers among the instrumental variables of genus *Hungatella*, genus *Haemophilus* and genus *Ruminococcaceae (UCG010)*. However, MR-PRESSO analysis indicated no outliers in the results (Supplementary Table S6). As a result, the available evidence did not support the presence of horizontal pleiotropy in the relationship between these bacteria and kidney stones.

### Differential abundance analysis

Differential abundance analysis of the gut microbiota revealed that, compared to the control group, the stone former group showed no significant difference in the abundance of *Oxalobacter formigenes*. The abundance of the genus *Anaerostipes*, genus *Bifidobacterium*, genus *Dialister*, genus *Erysipelotrichaceae_UCG-003*, genus *Muribaculaceae* and genus *Klebsiella* were decreased, while the abundance of genus *Acidaminococcus*, genus *Alloprevotella*, genus *Catenibacterium*, genus *Faecalitalea*, genus *Megasphaera*, genus *Parabacteroides*, genus *Prevotellaceae_NK3B31_group*, genus *Pseudomonas* and genus *Subdoligranulum* were increased in the stone former group. Additionally, there were some variations in the abundance of certain bacterial species within the genera *Bacteroides*, *Faecalibacterium*, and *Prevotella*, with some showing an increase and others showing a decrease (Supplementary Figure S3; Supplementary Tables S8, S9). The Pearson correlation coefficient for the correlation analysis is 0.694, indicating the presence of differences in gut microbiota between the stone former group and the control group.

## Discussion

As far as we are aware, this is the first MR analysis conducted to explore the potential causal relationship between gut microbiota and kidney stones. Neither MR analysis nor differential abundance analysis provide evidence of a causal association between genus *Oxalobacter*/*Oxalobacter formigenes* and kidney stones. Both MR analysis and differential abundance analysis showed genus *Subdoligranulum* were risk factors for kidney stones.

Calcium oxalate stones, comprising approximately 80% of all kidney stone varieties, represent the most commom classification of kidney stones (Khan et al., 2016). The gastrointestinal tract assumes a pivotal function in oxalate metabolism, as it exerts significant impact on the urinary oxalate excretion and, by extension, the proclivity for the development of kidney stones (Robijn et al., 2011). The existence of gut microbiota capable of degrading oxalate has the potential to impede oxalate absorption and reduce oxalate excretion. In recent decades, there has been a focused investigation into the contribution of gut microbiota to the physiopathologic of the gut–kidney axis. In fact, many of these studies have been predominantly centered on *Oxalobacter formigenes*, a type of oxalate-degrading bacteria *via* the expression of oxalyl-CoA decarboxylase and formyl-CoA transferase (Ellis et al., 2016). Several studies have provided evidence that patients with kidney stones have a lower incidence of *Oxalobacter formigenes* in their stool than healthy control subjects (Kumar et al., 2002, 2004; Kaufman et al., 2008). There also exists evidence indicating that patients with kidney stones who have *Oxalobacter formigenes* in their fecal matter experience a reduced level of urinary oxalate excretion in comparison to those normal individuals (Neuhaus et al., 2000; Kwak et al., 2003; Troxel et al., 2003). However, the conflicting results from both observational and intervention studies have created substantial uncertainty regarding the contribution of *Oxalobacter formigenes* to the formation of kidney stone (Ticinesi et al., 2019).

Numerous fact ors have been identified as being associated with the Oxalobacter colonization in the gut microbiota, including ethnicity, country of residence, age, education level, recent antibiotic usage, body weight, and nutritional imbalances (Liu M. et al., 2017; Ticinesi et al., 2020). By utilizing genomic shotgun data and shotgun community profiling analysis, a study showed a total of 31% of healthy young adults residing in the United States demonstrated a positive presence of *Oxalobacter formigenes*, which is much lower than that detected in tribal populations from Venezuela and Tanzania (Barnett et al., 2016; PeBenito et al., 2019). Some studies suggested lifetime exposure to antibiotics, especially long-duration treatments initiated at a younger age, is significantly associated with an increased risk of developing kidney stones (Tasian et al., 2018; Ferraro et al., 2019). A study indicated that prior exposure to antibiotics, even those that are not typically effective against *Oxalobacter formigenes*, can impact the colonization rates of this bacterium (Kaufman et al., 2008). Dietary habits and nutrition may constitute partial etiologies of the observed differences in gut microbiota composition between individuals with nephrolithiasis and the control group. Kaufman et al. have noted a positive correlation between oxalate consumption and the prevalence of *Oxalobacter formigenes* in the control group, due to the fact that dietary oxalate serves as a significant energy substrate for this bacterium, in addition to endogenously-produced oxalate (Kaufman et al., 2008). Intestinal oxalate absorption is also influenced by the ratio of dietary calcium to oxalate, and an optimal ratio promotes the formation of oxalate-calcium complexes in the gut lumen, thereby inhibiting intestinal absorption and promoting renal excretion, ultimately limiting the formation of kidney stones (Siener et al., 2003; Knight et al., 2006). In fact, most studies investigating the relationship between kidney stones and gut microbiota have not effectively integrated these factors. Furthermore, in a large case–control study on the relationship between *Oxalobacter formigenes* and kidney stone formation, no difference was observed in urinary oxalate excretion and the presence of *Oxalobacter formigenes* colonization (Kaufman et al., 2008).

In addition, two distinct studies have found significant correlations between the average relative abundance of certain specific taxa, including *Sutterella*, *Veillonella* and *Peptococcus*, and urinary oxalate excretion levels (Suryavanshi et al., 2016; Ticinesi et al., 2018), indicating that the gut-kidney axis may not solely depend on the presence of *Oxalobacter formigenes*. Subsequent investigations have indicated that, in healthy individuals, the presence of *Oxalobacter formigenes* is linked to an intricate network of bacteria that may possess oxalate-degrading capabilities themselves or promote the metabolic activity of *Oxalobacter formigenes* (Suryavanshi et al., 2018; Miller et al., 2019). This notion has been validated in mice that were transplanted with human fecal samples colonized by *Oxalobacter formigenes*, as the transplantation process led to a specific expansion of the bacteria network associated with *Oxalobacter formigenes* (Pebenito et al., 2019).

*Subdoligranulum* is a genus of anaerobic, spore-free Gram-negative bacteria. In studies investigating various diseases, genus *Subdoligranulum* has been consistently linked to chronic inflammation-related immune markers (Shi et al., 2021). Currently, it is widely believed that there is a close association between gut microbiota and obesity, as well as related metabolic diseases. A study has revealed that the abundance of genus *Subdoligranulum* is positively correlated with body weight and BMI (Kim et al., 2014). Li et al. conducted a study on the alterations in gut microbiota in individuals with hypertension and observed a positive correlation between the abundance of genus *Subdoligranulum* and both systolic and diastolic blood pressure (Li et al., 2019). Genus *Subdoligranulum* is also enriched in the diabetes mellitus individuals (Zhao et al., 2020). Therefore, the potential mechanisms may involve the involvement of gut microbiota in chronic inflammatory responses, as well as the mediation of obesity and metabolic dysregulation, which contribute to the formation of kidney stones.

Previous studies have indicated that certain bacterial genera, including *Prevotella*, *Acinetobacter*, *Pseudomonas*, *Staphylococcus*, *Megamonas*, and *Cetobacterium*, have been detected in individuals with kidney stones (Jandhyala et al., 2015; Liu et al., 2020). These genera are known to be associated with inflammatory diseases, implying that inflammation might play a role in the development of kidney stones. It is noteworthy that *Faecalibacterium* possesses the capability to synthesize short-chain fatty acids (SCFAs), particularly butyrate, which plays a crucial role in modulating inflammation. Hence, the depletion of this bacterium could contribute to the formation of kidney stones (Gambaro et al., 2017).

This study has several notable strengths. Firstly, previous case–control studies were unable to establish the temporal sequence between gut microbiota colonization and kidney stone formation, as stool samples were collected after episode occurred. While the MR analysis is an appropriate method for investigating causal relationships, as it can eliminate potential confounding factors and reverse causation, and differential abundance analysis helps improve the stability of our results. Secondly, the genetic variants of gut microbiota used in the study were obtained from the largest available GWAS meta-analysis, which ensured the strength and reliability of the instruments used in the MR analysis. Thirdly, horizontal pleiotropy was detected and addressed through the use of MR-PRESSO and MR-Egger regression intercept term tests.

The present study also has some limitations. Firstly, since the exposure dataset only provided information at the genus taxonomic level, we were unable to investigate the causal association between gut microbiota and kidney stones at the species level. Secondly, kidney stone composition was not available in the FinnGen consortium, thereby impeding our ability to conduct subgroup analyses. Further studies with subgroup analysis stratified by the composition of kidney stones may yield more reliable results. Thirdly, although the majority of participants in the GWAS meta-analysis for gut microbiota data were of European ancestry, there is still potential for population stratification bias, and the findings of this study may not be fully generalizable to individuals of non-European ancestry. Finally, We were unable to conduct correlation analyses of gut microbiota abundance between kidney stone patients and healthy individuals.

## Conclusion

Our two-sample MR study did not find any causal relationship between genus *Oxalobacter* and kidney stones. The association between gut microbiota and kidney stones does not solely depend on the presence of *Oxalobacter formigenes*. A more integrated approach using multiple omics platforms is needed to better understand the pathogenesis of kidney stones in the context of complex gene–environment interactions over time.

## Data availability statement

The original contributions presented in the study are included in the article/Supplementary material, further inquiries can be directed to the corresponding authors.

## Ethics statement

The patients/participants provided their written informed consent to participate in this study.

## Author contributions

ML: project development, data analysis, and manuscript writing. YZ, JW, and MG: data collection and analysis. YZ: data collection. JW and MG: manuscript editing. ZZ and HC: project development and manuscript editing. All authors contributed to the article and approved the submitted version.

## Funding

Funding was provided by the Fundamental Research Funds for the Central Universities of Central South University (2022ZZTS0294 to ML; 2021zzts0348 to ZZ), the National Natural Science Foundation of China (82170781 to HC) and Natural Science Foundation of Hunan Province (2021JJ31050 to HC).

## Conflict of interest

The authors declare that the research was conducted in the absence of any commercial or financial relationships that could be construed as a potential conflict of interest.

## Publisher’s note

All claims expressed in this article are solely those of the authors and do not necessarily represent those of their affiliated organizations, or those of the publisher, the editors and the reviewers. Any product that may be evaluated in this article, or claim that may be made by its manufacturer, is not guaranteed or endorsed by the publisher.

## Footnotes

^1^http://www.phenoscanner.medschl.cam.ac.uk/

## Abbreviations

MR: Mendelian randomization
RCTs: randomized controlled trials
SNPs: single-nucleotide polymorphisms
GWASs: genome-wide association studies
IVW: inverse-variance weighted
