# Supplementary material for: Causal relationship between kidney stones and gut microbiota contributes to the gut-kidney axis: a two-sample Mendelian randomization study
Source: Front Microbiol. 2023 Jul 12;14:1204311. doi: 10.3389/fmicb.2023.1204311 (PMC10368867; doi:10.3389/fmicb.2023.1204311)
Supplement: Supplementary file 1 [file Data_Sheet_1.ZIP › Suppl.Image&Table/Table S1-S6.docx]

**Table S1 Instrumental variables used in MR analysis of the association between gut microbiota and kidney stones.**

| **Group** | **Gut microbiota** | **SNP** | **Effect allele** | **Other allele** | **Exposure (Bacteria)** | | | **Outcome (Urolithiasis)** | | |
| --- | --- | --- | --- | --- | --- | --- | --- | --- | --- | --- |
|  |  |  |  |  | **Beta** | **SE** | **P-value** | **Beta** | **SE** | **P-value** |
| Order | Actinomycetales | rs35011108 | G | A | 0.242 | 0.050 | 1.88E-06 | -0.056 | 0.034 | 0.096 |
|  |  | rs2889192 | T | G | 0.088 | 0.019 | 3.97E-06 | 0.043 | 0.024 | 0.070 |
|  |  | rs34583783 | T | G | 0.124 | 0.026 | 5.54E-06 | -0.040 | 0.035 | 0.257 |
|  |  | rs4073240 | A | G | 0.075 | 0.016 | 5.68E-06 | -0.001 | 0.017 | 0.958 |
| Family | Actinomycetaceae | rs35011108 | G | A | 0.242 | 0.050 | 1.83E-06 | -0.056 | 0.034 | 0.096 |
|  |  | rs2889192 | T | G | 0.089 | 0.020 | 3.64E-06 | 0.043 | 0.024 | 0.070 |
|  |  | rs34583783 | T | G | 0.124 | 0.026 | 5.48E-06 | -0.040 | 0.035 | 0.257 |
|  |  | rs4073240 | A | G | 0.075 | 0.016 | 6.05E-06 | -0.001 | 0.017 | 0.958 |
| Family | Clostridiaceae 1 | rs12186080 | A | G | 0.075 | 0.016 | 5.34E-06 | -0.008 | 0.023 | 0.730 |
|  |  | rs2795528 | A | G | -0.181 | 0.039 | 3.81E-06 | 0.023 | 0.036 | 0.528 |
|  |  | rs550843 | C | T | -0.073 | 0.017 | 7.09E-06 | 0.014 | 0.019 | 0.442 |
|  |  | rs12341505 | A | G | 0.081 | 0.018 | 4.54E-06 | -0.015 | 0.029 | 0.613 |
|  |  | rs4723021 | C | T | -0.106 | 0.024 | 7.42E-06 | 0.052 | 0.033 | 0.123 |
|  |  | rs881532 | G | A | -0.053 | 0.012 | 7.90E-06 | -0.002 | 0.017 | 0.913 |
|  |  | rs2817172 | T | C | 0.056 | 0.012 | 5.27E-06 | -0.037 | 0.017 | 0.030 |
|  |  | rs10875374 | T | C | -0.054 | 0.012 | 8.10E-06 | 0.012 | 0.017 | 0.488 |
|  |  | rs62397761 | G | A | 0.062 | 0.014 | 9.08E-06 | -0.012 | 0.018 | 0.503 |
|  |  | rs56188186 | G | A | 0.097 | 0.022 | 8.24E-06 | -0.086 | 0.039 | 0.029 |
| Genus | Clostridiumsensustricto 1 | rs550843 | C | T | -0.078 | 0.017 | 2.05E-06 | 0.442 | 0.014 | 0.019 |
|  |  | rs2795528 | A | G | -0.184 | 0.039 | 2.72E-06 | 0.528 | 0.023 | 0.036 |
|  |  | rs2817172 | T | C | 0.058 | 0.012 | 2.77E-06 | 0.030 | -0.037 | 0.017 |
|  |  | rs115807074 | G | A | -0.227 | 0.049 | 4.32E-06 | 0.704 | 0.031 | 0.081 |
|  |  | rs116847295 | T | C | 0.110 | 0.025 | 4.58E-06 | 0.057 | -0.048 | 0.025 |
|  |  | rs12341505 | A | G | 0.081 | 0.018 | 4.82E-06 | 0.613 | -0.015 | 0.029 |
|  |  | rs11264403 | A | G | -0.139 | 0.033 | 7.76E-06 | 0.654 | -0.014 | 0.032 |
| Genus | Haemophilus | rs9382510 | T | C | -0.094 | 0.017 | 7.12E-08 | -0.027 | 0.019 | 0.160 |
|  |  | rs76022354 | T | C | 0.245 | 0.051 | 1.83E-06 | 0.032 | 0.038 | 0.403 |
|  |  | rs111582866 | A | G | -0.124 | 0.026 | 1.27E-06 | -0.007 | 0.030 | 0.805 |
|  |  | rs9895850 | C | T | -0.193 | 0.042 | 2.14E-06 | -0.005 | 0.041 | 0.907 |
|  |  | rs35509 | A | G | 0.128 | 0.027 | 2.01E-06 | -0.026 | 0.042 | 0.536 |
|  |  | rs78909003 | C | T | -0.246 | 0.050 | 1.67E-06 | -0.051 | 0.037 | 0.165 |
|  |  | rs9328464 | C | T | 0.072 | 0.015 | 1.42E-06 | 0.005 | 0.017 | 0.786 |
|  |  | rs4822728 | C | T | 0.071 | 0.015 | 3.48E-06 | 0.037 | 0.017 | 0.027 |
|  |  | rs10781340 | A | G | 0.095 | 0.020 | 4.32E-06 | 0.004 | 0.025 | 0.867 |
| Genus | Hungatella | rs13128780 | C | T | -0.150 | 0.031 | 1.75E-06 | 0.021 | 0.021 | 0.326 |
|  |  | rs72759041 | T | G | -0.126 | 0.028 | 3.86E-06 | 0.022 | 0.021 | 0.292 |
|  |  | rs10044993 | A | C | 0.140 | 0.032 | 8.07E-06 | 0.023 | 0.030 | 0.448 |
|  |  | rs13249325 | G | T | -0.100 | 0.023 | 9.69E-06 | 0.024 | 0.017 | 0.148 |
|  |  | rs17092615 | A | G | 0.152 | 0.034 | 7.38E-06 | -0.009 | 0.024 | 0.710 |
| Genus | Ruminococcaceae (UCG010) | rs682403 | G | A | -0.059 | 0.012 | 2.37E-06 | -0.056 | 0.017 | 0.001 |
|  |  | rs6958419 | T | C | -0.059 | 0.012 | 2.84E-06 | -0.012 | 0.017 | 0.463 |
|  |  | rs2820282 | C | A | -0.059 | 0.013 | 2.85E-06 | -0.013 | 0.017 | 0.463 |
|  |  | rs12597105 | A | G | 0.067 | 0.014 | 4.87E-06 | -0.021 | 0.021 | 0.334 |
|  |  | rs73218807 | A | G | -0.166 | 0.037 | 6.43E-06 | -0.039 | 0.031 | 0.204 |
|  |  | rs7441445 | T | C | -0.057 | 0.013 | 6.80E-06 | 0.031 | 0.017 | 0.063 |
|  |  |  |  |  | -0.051 | 0.011 | 2.10E-06 | -0.009 | 0.017 | 0.584 |
| Genus | Subdoligranulum | rs10065321 | C | T | 0.166 | 0.036 | 2.18E-06 | 0.003 | 0.047 | 0.955 |
|  |  | rs4347804 | G | A | -0.074 | 0.016 | 2.81E-06 | -0.006 | 0.024 | 0.815 |
|  |  | rs6555306 | C | T | -0.054 | 0.012 | 3.87E-06 | 0.050 | 0.019 | 0.010 |
|  |  | rs3761728 | G | T | -0.104 | 0.023 | 2.72E-06 | -0.037 | 0.026 | 0.158 |
|  |  | rs2114677 | T | C | -0.072 | 0.016 | 7.52E-06 | 0.002 | 0.024 | 0.923 |
|  |  | rs75158211 | C | T | 0.107 | 0.023 | 4.51E-06 | 0.027 | 0.032 | 0.405 |
|  |  | rs2171249 | T | C | -0.051 | 0.011 | 4.22E-06 | -0.007 | 0.018 | 0.696 |
|  |  | rs35940633 | A | G | -0.143 | 0.031 | 7.41E-06 | -0.023 | 0.030 | 0.455 |
|  |  | rs76528319 | T | G | 0.049 | 0.011 | 6.72E-06 | 0.012 | 0.017 | 0.471 |
|  |  | rs1667315 | A | G | -0.052 | 0.012 | 8.38E-06 | 0.046 | 0.020 | 0.024 |
|  |  | rs10497836 | T | C | 0.242 | 0.050 | 1.88E-06 | -0.056 | 0.034 | 0.096 |

**Table S2 Full** **MR results of causal links between gut microbiome and kidney stones risk.**

| **Group** | **Gut microbiota** | **MR method** | **No.SNP** | **OR (95% CI)** | **P-value** |
| --- | --- | --- | --- | --- | --- |
| Phylum | Actinomycetota | MR Egger | 14 | 2.90 (1.43-5.90) | 0.012 |
|  |  | Weighted median | 14 | 1.01 (0.78-1.31) | 0.951 |
|  |  | Inverse variance weighted | 14 | 1.11 (0.92-1.36) | 0.277 |
|  |  | Simple mode | 14 | 0.91 (0.54-1.54) | 0.736 |
|  |  | Weighted mode | 14 | 0.91 (0.52-1.60) | 0.743 |
| Phylum | Bacteroidota | MR Egger | 10 | 0.88 (0.56-1.38) | 0.598 |
|  |  | Weighted median | 10 | 0.91 (0.70-1.17) | 0.454 |
|  |  | Inverse variance weighted | 10 | 0.90 (0.74-1.10) | 0.314 |
|  |  | Simple mode | 10 | 0.91 (0.64-1.30) | 0.625 |
|  |  | Weighted mode | 10 | 0.90 (0.64-1.28) | 0.585 |
| Phylum | Cyanobacteriota | MR Egger | 8 | 1.55 (0.85-2.82) | 0.204 |
|  |  | Weighted median | 8 | 1.00 (0.81-1.24) | 0.967 |
|  |  | Inverse variance weighted | 8 | 0.96 (0.80-1.16) | 0.671 |
|  |  | Simple mode | 8 | 1.06 (0.75-1.49) | 0.763 |
|  |  | Weighted mode | 8 | 1.05 (0.78-1.39) | 0.771 |
| Phylum | Euryarchaeota | MR Egger | 11 | 1.23 (0.82-1.84) | 0.351 |
|  |  | Weighted median | 11 | 1.11 (0.98-1.26) | 0.102 |
|  |  | Inverse variance weighted | 11 | 1.06 (0.97-1.16) | 0.210 |
|  |  | Simple mode | 11 | 1.13 (0.91-1.40) | 0.296 |
|  |  | Weighted mode | 11 | 1.13 (0.92-1.38) | 0.263 |
| Phylum | Bacillota | MR Egger | 14 | 0.99 (0.59-1.66) | 0.960 |
|  |  | Weighted median | 14 | 1.01 (0.80-1.28) | 0.926 |
|  |  | Inverse variance weighted | 14 | 0.87 (0.72-1.05) | 0.147 |
|  |  | Simple mode | 14 | 1.10 (0.74-1.66) | 0.641 |
|  |  | Weighted mode | 14 | 1.09 (0.78-1.52) | 0.634 |
| Phylum | Lentisphaerota | MR Egger | 9 | 0.79 (0.50-1.26) | 0.356 |
|  |  | Weighted median | 9 | 1.02 (0.87-1.20) | 0.792 |
|  |  | Inverse variance weighted | 9 | 1.00 (0.88-1.13) | 0.954 |
|  |  | Simple mode | 9 | 1.03 (0.81-1.30) | 0.823 |
|  |  | Weighted mode | 9 | 1.03 (0.81-1.30) | 0.821 |
| Phylum | Pseudomonadota | MR Egger | 12 | 1.03 (0.55-1.93) | 0.938 |
|  |  | Weighted median | 12 | 0.96 (0.74-1.25) | 0.751 |
|  |  | Inverse variance weighted | 12 | 1.03 (0.83-1.27) | 0.818 |
|  |  | Simple mode | 12 | 0.87 (0.51-1.48) | 0.613 |
|  |  | Weighted mode | 12 | 0.81 (0.49-1.32) | 0.412 |
| Phylum | Mycoplasmatota | MR Egger | 12 | 0.80 (0.48-1.33) | 0.408 |
|  |  | Weighted median | 12 | 0.92 (0.76-1.11) | 0.394 |
|  |  | Inverse variance weighted | 12 | 0.94 (0.81-1.09) | 0.398 |
|  |  | Simple mode | 12 | 0.97 (0.71-1.32) | 0.829 |
|  |  | Weighted mode | 12 | 0.94 (0.70-1.26) | 0.704 |
| Phylum | Verrucomicrobia | MR Egger | 12 | 1.07 (0.72-1.59) | 0.738 |
|  |  | Weighted median | 12 | 0.98 (0.80-1.20) | 0.836 |
|  |  | Inverse variance weighted | 12 | 1.05 (0.91-1.22) | 0.489 |
|  |  | Simple mode | 12 | 0.93 (0.66-1.30) | 0.671 |
|  |  | Weighted mode | 12 | 0.92 (0.67-1.27) | 0.622 |
|  |  |  |  |  |  |
| Class | Actinomycetes | MR Egger | 14 | 1.43 (0.89-2.29) | 0.170 |
|  |  | Weighted median | 14 | 1.11 (0.89-1.39) | 0.347 |
|  |  | Inverse variance weighted | 14 | 1.17 (0.99-1.38) | 0.065 |
|  |  | Simple mode | 14 | 1.09 (0.73-1.62) | 0.680 |
|  |  | Weighted mode | 14 | 1.17 (0.83-1.64) | 0.378 |
| Class | Alphaproteobacteria | MR Egger | 7 | 1.27 (0.63-2.54) | 0.534 |
|  |  | Weighted median | 7 | 1.10 (0.87-1.39) | 0.412 |
|  |  | Inverse variance weighted | 7 | 1.02 (0.86-1.22) | 0.817 |
|  |  | Simple mode | 7 | 1.17 (0.81-1.68) | 0.429 |
|  |  | Weighted mode | 7 | 1.16 (0.85-1.59) | 0.391 |
| Class | Bacilli | MR Egger | 18 | 0.73 (0.49-1.10) | 0.154 |
|  |  | Weighted median | 18 | 1.07 (0.88-1.30) | 0.515 |
|  |  | Inverse variance weighted | 18 | 1.03 (0.89-1.19) | 0.676 |
|  |  | Simple mode | 18 | 1.16 (0.81-1.66) | 0.431 |
|  |  | Weighted mode | 18 | 1.15 (0.80-1.67) | 0.464 |
| Class | Bacteroidia | MR Egger | 13 | 0.91 (0.60-1.39) | 0.668 |
|  |  | Weighted median | 13 | 0.92 (0.74-1.15) | 0.477 |
|  |  | Inverse variance weighted | 13 | 0.90 (0.75-1.08) | 0.253 |
|  |  | Simple mode | 13 | 0.94 (0.67-1.32) | 0.713 |
|  |  | Weighted mode | 13 | 0.94 (0.67-1.31) | 0.706 |
| Class | Betaproteobacteria | MR Egger | 10 | 1.27 (0.68-2.40) | 0.475 |
|  |  | Weighted median | 10 | 1.16 (0.89-1.50) | 0.268 |
|  |  | Inverse variance weighted | 10 | 1.11 (0.91-1.36) | 0.291 |
|  |  | Simple mode | 10 | 1.18 (0.76-1.83) | 0.477 |
|  |  | Weighted mode | 10 | 1.20 (0.79-1.82) | 0.420 |
| Class | Clostridia | MR Egger | 10 | 1.01 (0.36-2.89) | 0.980 |
|  |  | Weighted median | 10 | 0.84 (0.64-1.11) | 0.217 |
|  |  | Inverse variance weighted | 10 | 0.83 (0.66-1.04) | 0.110 |
|  |  | Simple mode | 10 | 0.83 (0.53-1.29) | 0.430 |
|  |  | Weighted mode | 10 | 0.90 (0.62-1.33) | 0.620 |
| Class | Coriobacteriia | MR Egger | 13 | 0.89 (0.39-2.07) | 0.797 |
|  |  | Weighted median | 13 | 0.94 (0.73-1.22) | 0.645 |
|  |  | Inverse variance weighted | 13 | 0.96 (0.79-1.16) | 0.667 |
|  |  | Simple mode | 13 | 1.01 (0.67-1.53) | 0.958 |
|  |  | Weighted mode | 13 | 0.96 (0.66-1.42) | 0.853 |
| Class | Deltaproteobacteria | MR Egger | 11 | 0.87 (0.24-3.14) | 0.839 |
|  |  | Weighted median | 11 | 1.03 (0.78-1.36) | 0.822 |
|  |  | Inverse variance weighted | 11 | 0.89 (0.71-1.13) | 0.351 |
|  |  | Simple mode | 11 | 1.16 (0.72-1.88) | 0.553 |
|  |  | Weighted mode | 11 | 1.16 (0.72-1.87) | 0.549 |
| Class | Erysipelotrichia | MR Egger | 13 | 1.90 (0.84-4.31) | 0.153 |
|  |  | Weighted median | 13 | 1.08 (0.84-1.41) | 0.542 |
|  |  | Inverse variance weighted | 13 | 1.02 (0.84-1.23) | 0.876 |
|  |  | Simple mode | 13 | 1.34 (0.82-2.21) | 0.269 |
|  |  | Weighted mode | 13 | 1.36 (0.83-2.22) | 0.247 |
| Class | Gammaproteobacteria | MR Egger | 7 | 0.58 (0.15-2.28) | 0.470 |
|  |  | Weighted median | 7 | 0.73 (0.51-1.04) | 0.083 |
|  |  | Inverse variance weighted | 7 | 0.89 (0.59-1.33) | 0.561 |
|  |  | Simple mode | 7 | 0.67 (0.34-1.34) | 0.299 |
|  |  | Weighted mode | 7 | 0.65 (0.39-1.07) | 0.143 |
| Class | Lentisphaeria | MR Egger | 8 | 0.80 (0.48-1.32) | 0.415 |
|  |  | Weighted median | 8 | 0.99 (0.84-1.17) | 0.914 |
|  |  | Inverse variance weighted | 8 | 0.99 (0.86-1.14) | 0.912 |
|  |  | Simple mode | 8 | 1.02 (0.77-1.35) | 0.879 |
|  |  | Weighted mode | 8 | 1.02 (0.78-1.31) | 0.912 |
| Class | Melainabacteria | MR Egger | 10 | 1.04 (0.72-1.50) | 0.833 |
|  |  | Weighted median | 10 | 0.96 (0.82-1.13) | 0.633 |
|  |  | Inverse variance weighted | 10 | 0.90 (0.80-1.01) | 0.081 |
|  |  | Simple mode | 10 | 0.97 (0.77-1.23) | 0.830 |
|  |  | Weighted mode | 10 | 0.98 (0.78-1.23) | 0.857 |
| Class | Methanobacteria | MR Egger | 9 | 1.18 (0.78-1.78) | 0.450 |
|  |  | Weighted median | 9 | 0.96 (0.84-1.11) | 0.609 |
|  |  | Inverse variance weighted | 9 | 0.99 (0.89-1.10) | 0.880 |
|  |  | Simple mode | 9 | 0.88 (0.70-1.12) | 0.342 |
|  |  | Weighted mode | 9 | 0.93 (0.75-1.16) | 0.537 |
| Class | Mollicutes | MR Egger | 12 | 0.80 (0.48-1.33) | 0.408 |
|  |  | Weighted median | 12 | 0.92 (0.76-1.12) | 0.403 |
|  |  | Inverse variance weighted | 12 | 0.94 (0.81-1.09) | 0.398 |
|  |  | Simple mode | 12 | 0.97 (0.71-1.31) | 0.826 |
|  |  | Weighted mode | 12 | 0.94 (0.71-1.26) | 0.698 |
| Class | Negativicutes | MR Egger | 12 | 1.00 (0.52-1.90) | 0.989 |
|  |  | Weighted median | 12 | 0.94 (0.73-1.20) | 0.602 |
|  |  | Inverse variance weighted | 12 | 0.97 (0.80-1.17) | 0.760 |
|  |  | Simple mode | 12 | 0.85 (0.57-1.27) | 0.450 |
|  |  | Weighted mode | 12 | 0.86 (0.57-1.29) | 0.476 |
| Class | Verrucomicrobiae | MR Egger | 11 | 1.36 (0.80-2.31) | 0.281 |
|  |  | Weighted median | 11 | 0.91 (0.74-1.12) | 0.369 |
|  |  | Inverse variance weighted | 11 | 0.96 (0.82-1.12) | 0.620 |
|  |  | Simple mode | 11 | 0.84 (0.60-1.19) | 0.350 |
|  |  | Weighted mode | 11 | 0.85 (0.59-1.24) | 0.421 |
|  |  |  |  |  |  |
| Order | Actinomycetales | MR Egger | 4 | 0.75 (0.48-1.16) | 0.322 |
|  |  | Weighted median | 4 | 0.79 (0.61-1.02) | 0.066 |
|  |  | Inverse variance weighted | 4 | 0.79 (0.65-0.96) | 0.020 |
|  |  | Simple mode | 4 | 0.74 (0.52-1.04) | 0.178 |
|  |  | Weighted mode | 4 | 0.79 (0.60-1.03) | 0.183 |
| Order | Bacillales | MR Egger | 8 | 1.01 (0.66-1.56) | 0.954 |
|  |  | Weighted median | 8 | 1.05 (0.92-1.20) | 0.458 |
|  |  | Inverse variance weighted | 8 | 1.01 (0.91-1.12) | 0.845 |
|  |  | Simple mode | 8 | 1.09 (0.90-1.31) | 0.398 |
|  |  | Weighted mode | 8 | 1.08 (0.88-1.32) | 0.504 |
| Order | Bacteroidales | MR Egger | 13 | 0.91 (0.60-1.39) | 0.668 |
|  |  | Weighted median | 13 | 0.92 (0.72-1.17) | 0.505 |
|  |  | Inverse variance weighted | 13 | 0.90 (0.75-1.08) | 0.253 |
|  |  | Simple mode | 13 | 0.94 (0.66-1.33) | 0.721 |
|  |  | Weighted mode | 13 | 0.94 (0.67-1.31) | 0.710 |
| Order | Bifidobacteriales | MR Egger | 11 | 1.82 (1.08-3.05) | 0.051 |
|  |  | Weighted median | 11 | 1.16 (0.91-1.47) | 0.226 |
|  |  | Inverse variance weighted | 11 | 1.17 (0.98-1.38) | 0.079 |
|  |  | Simple mode | 11 | 1.08 (0.71-1.64) | 0.731 |
|  |  | Weighted mode | 11 | 1.11 (0.83-1.49) | 0.502 |
| Order | Burkholderiales | MR Egger | 10 | 1.28 (0.69-2.39) | 0.458 |
|  |  | Weighted median | 10 | 1.07 (0.82-1.39) | 0.616 |
|  |  | Inverse variance weighted | 10 | 1.03 (0.85-1.26) | 0.763 |
|  |  | Simple mode | 10 | 1.14 (0.74-1.76) | 0.561 |
|  |  | Weighted mode | 10 | 1.17 (0.74-1.83) | 0.520 |
| Order | Eubacteriales | MR Egger | 11 | 1.10 (0.42-2.87) | 0.846 |
|  |  | Weighted median | 11 | 0.88 (0.67-1.16) | 0.357 |
|  |  | Inverse variance weighted | 11 | 0.83 (0.66-1.03) | 0.095 |
|  |  | Simple mode | 11 | 0.91 (0.58-1.45) | 0.709 |
|  |  | Weighted mode | 11 | 0.95 (0.64-1.40) | 0.795 |
| Order | Coriobacteriales | MR Egger | 13 | 0.89 (0.39-2.07) | 0.797 |
|  |  | Weighted median | 13 | 0.94 (0.73-1.22) | 0.646 |
|  |  | Inverse variance weighted | 13 | 0.96 (0.79-1.16) | 0.667 |
|  |  | Simple mode | 13 | 1.01 (0.68-1.51) | 0.956 |
|  |  | Weighted mode | 13 | 0.96 (0.67-1.38) | 0.841 |
| Order | Desulfovibrionales | MR Egger | 10 | 0.82 (0.21-3.12) | 0.776 |
|  |  | Weighted median | 10 | 1.05 (0.79-1.39) | 0.732 |
|  |  | Inverse variance weighted | 10 | 0.92 (0.71-1.18) | 0.500 |
|  |  | Simple mode | 10 | 1.19 (0.81-1.77) | 0.402 |
|  |  | Weighted mode | 10 | 1.18 (0.77-1.81) | 0.462 |
| Order | Enterobacterales | MR Egger | 7 | 0.31 (0.02-5.87) | 0.473 |
|  |  | Weighted median | 7 | 1.29 (0.87-1.92) | 0.204 |
|  |  | Inverse variance weighted | 7 | 1.09 (0.68-1.77) | 0.711 |
|  |  | Simple mode | 7 | 1.40 (0.61-3.19) | 0.456 |
|  |  | Weighted mode | 7 | 1.41 (0.70-2.84) | 0.374 |
| Order | Erysipelotrichales | MR Egger | 13 | 1.90 (0.84-4.31) | 0.153 |
|  |  | Weighted median | 13 | 1.08 (0.83-1.41) | 0.546 |
|  |  | Inverse variance weighted | 13 | 1.02 (0.84-1.23) | 0.876 |
|  |  | Simple mode | 13 | 1.34 (0.81-2.22) | 0.274 |
|  |  | Weighted mode | 13 | 1.36 (0.83-2.22) | 0.248 |
| Order | Candidatus Gastranaerophilales | MR Egger | 9 | 1.05 (0.72-1.54) | 0.805 |
|  |  | Weighted median | 9 | 0.98 (0.83-1.16) | 0.823 |
|  |  | Inverse variance weighted | 9 | 0.90 (0.79-1.02) | 0.107 |
|  |  | Simple mode | 9 | 1.00 (0.77-1.29) | 0.972 |
|  |  | Weighted mode | 9 | 1.00 (0.80-1.25) | 0.994 |
| Order | Lactobacillales | MR Egger | 15 | 0.76 (0.50-1.16) | 0.226 |
|  |  | Weighted median | 15 | 0.98 (0.79-1.22) | 0.880 |
|  |  | Inverse variance weighted | 15 | 1.01 (0.86-1.19) | 0.871 |
|  |  | Simple mode | 15 | 0.96 (0.66-1.39) | 0.820 |
|  |  | Weighted mode | 15 | 0.95 (0.68-1.33) | 0.777 |
| Order | Methanobacteriales | MR Egger | 9 | 1.18 (0.78-1.78) | 0.450 |
|  |  | Weighted median | 9 | 0.96 (0.84-1.11) | 0.620 |
|  |  | Inverse variance weighted | 9 | 0.99 (0.89-1.10) | 0.880 |
|  |  | Simple mode | 9 | 0.88 (0.70-1.12) | 0.328 |
|  |  | Weighted mode | 9 | 0.93 (0.73-1.18) | 0.561 |
| Order | Mollicutes | MR Egger | 12 | 1.04 (0.67-1.61) | 0.864 |
|  |  | Weighted median | 12 | 1.02 (0.84-1.24) | 0.841 |
|  |  | Inverse variance weighted | 12 | 1.02 (0.89-1.18) | 0.757 |
|  |  | Simple mode | 12 | 1.18 (0.86-1.62) | 0.333 |
|  |  | Weighted mode | 12 | 1.06 (0.80-1.42) | 0.686 |
| Order | NB1n | MR Egger | 12 | 1.04 (0.59-1.82) | 0.899 |
|  |  | Weighted median | 12 | 0.90 (0.79-1.04) | 0.155 |
|  |  | Inverse variance weighted | 12 | 0.92 (0.80-1.05) | 0.212 |
|  |  | Simple mode | 12 | 0.85 (0.68-1.07) | 0.201 |
|  |  | Weighted mode | 12 | 0.87 (0.70-1.08) | 0.222 |
| Order | Pasteurellales | MR Egger | 13 | 0.95 (0.70-1.28) | 0.728 |
|  |  | Weighted median | 13 | 1.09 (0.92-1.29) | 0.317 |
|  |  | Inverse variance weighted | 13 | 1.08 (0.94-1.25) | 0.275 |
|  |  | Simple mode | 13 | 1.09 (0.84-1.43) | 0.515 |
|  |  | Weighted mode | 13 | 1.09 (0.88-1.34) | 0.433 |
| Order | Rhodospirillales | MR Egger | 14 | 0.81 (0.50-1.30) | 0.395 |
|  |  | Weighted median | 14 | 1.07 (0.91-1.26) | 0.385 |
|  |  | Inverse variance weighted | 14 | 1.09 (0.97-1.22) | 0.167 |
|  |  | Simple mode | 14 | 1.12 (0.85-1.49) | 0.432 |
|  |  | Weighted mode | 14 | 0.95 (0.73-1.25) | 0.730 |
| Order | Selenomonadales | MR Egger | 12 | 1.00 (0.52-1.90) | 0.989 |
|  |  | Weighted median | 12 | 0.94 (0.74-1.19) | 0.589 |
|  |  | Inverse variance weighted | 12 | 0.97 (0.80-1.17) | 0.760 |
|  |  | Simple mode | 12 | 0.85 (0.58-1.25) | 0.432 |
|  |  | Weighted mode | 12 | 0.86 (0.57-1.30) | 0.482 |
| Order | Verrucomicrobiales | MR Egger | 11 | 1.36 (0.80-2.31) | 0.281 |
|  |  | Weighted median | 11 | 0.91 (0.74-1.13) | 0.386 |
|  |  | Inverse variance weighted | 11 | 0.96 (0.82-1.12) | 0.620 |
|  |  | Simple mode | 11 | 0.84 (0.59-1.21) | 0.373 |
|  |  | Weighted mode | 11 | 0.85 (0.57-1.27) | 0.448 |
| Order | Victivallales | MR Egger | 8 | 0.80 (0.48-1.32) | 0.415 |
|  |  | Weighted median | 8 | 0.99 (0.84-1.17) | 0.914 |
|  |  | Inverse variance weighted | 8 | 0.99 (0.86-1.14) | 0.912 |
|  |  | Simple mode | 8 | 1.02 (0.79-1.33) | 0.871 |
|  |  | Weighted mode | 8 | 1.02 (0.79-1.30) | 0.909 |
| Family | Acidaminococcaceae | MR Egger | 7 | 0.94 (0.53-1.65) | 0.831 |
|  |  | Weighted median | 7 | 1.09 (0.86-1.38) | 0.495 |
|  |  | Inverse variance weighted | 7 | 1.06 (0.88-1.27) | 0.547 |
|  |  | Simple mode | 7 | 1.13 (0.82-1.56) | 0.489 |
|  |  | Weighted mode | 7 | 1.09 (0.82-1.45) | 0.568 |
| Family | Actinomycetaceae | MR Egger | 4 | 0.75 (0.48-1.15) | 0.320 |
|  |  | Weighted median | 4 | 0.79 (0.61-1.01) | 0.058 |
|  |  | Inverse variance weighted | 4 | 0.79 (0.65-0.96) | 0.019 |
|  |  | Simple mode | 4 | 0.74 (0.52-1.05) | 0.188 |
|  |  | Weighted mode | 4 | 0.79 (0.60-1.03) | 0.180 |
| Family | Alcaligenaceae | MR Egger | 11 | 1.10 (0.45-2.73) | 0.836 |
|  |  | Weighted median | 11 | 1.06 (0.82-1.36) | 0.649 |
|  |  | Inverse variance weighted | 11 | 1.05 (0.86-1.28) | 0.648 |
|  |  | Simple mode | 11 | 1.07 (0.70-1.65) | 0.756 |
|  |  | Weighted mode | 11 | 1.08 (0.73-1.58) | 0.716 |
| Family | Bacteroidaceae | MR Egger | 7 | 0.63 (0.12-3.30) | 0.609 |
|  |  | Weighted median | 7 | 1.24 (0.87-1.76) | 0.231 |
|  |  | Inverse variance weighted | 7 | 1.23 (0.89-1.70) | 0.210 |
|  |  | Simple mode | 7 | 1.50 (0.79-2.85) | 0.261 |
|  |  | Weighted mode | 7 | 1.41 (0.74-2.72) | 0.338 |
| Family | Muribaculaceae | MR Egger | 8 | 1.21 (0.67-2.17) | 0.556 |
|  |  | Weighted median | 8 | 1.08 (0.89-1.32) | 0.449 |
|  |  | Inverse variance weighted | 8 | 0.95 (0.82-1.10) | 0.527 |
|  |  | Simple mode | 8 | 1.09 (0.79-1.50) | 0.629 |
|  |  | Weighted mode | 8 | 1.09 (0.82-1.45) | 0.553 |
| Family | Bifidobacteriaceae | MR Egger | 11 | 1.82 (1.08-3.05) | 0.051 |
|  |  | Weighted median | 11 | 1.16 (0.92-1.46) | 0.214 |
|  |  | Inverse variance weighted | 11 | 1.17 (0.98-1.38) | 0.079 |
|  |  | Simple mode | 11 | 1.08 (0.70-1.66) | 0.735 |
|  |  | Weighted mode | 11 | 1.11 (0.79-1.56) | 0.561 |
| Family | Christensenellaceae | MR Egger | 11 | 1.23 (0.92-1.64) | 0.203 |
|  |  | Weighted median | 11 | 1.25 (1.02-1.54) | 0.032 |
|  |  | Inverse variance weighted | 11 | 1.14 (0.98-1.32) | 0.092 |
|  |  | Simple mode | 11 | 1.33 (0.91-1.95) | 0.172 |
|  |  | Weighted mode | 11 | 1.31 (0.97-1.77) | 0.107 |
| Family | Clostridiaceae 1 | MR Egger | 10 | 0.73 (0.43-1.24) | 0.276 |
|  |  | Weighted median | 10 | 0.84 (0.66-1.08) | 0.184 |
|  |  | Inverse variance weighted | 10 | 0.80 (0.67-0.96) | 0.015 |
|  |  | Simple mode | 10 | 0.86 (0.61-1.22) | 0.430 |
|  |  | Weighted mode | 10 | 0.86 (0.63-1.18) | 0.386 |
| Family | Clostridialesvadin (BB60 group) | MR Egger | 15 | 1.16 (0.82-1.64) | 0.414 |
|  |  | Weighted median | 15 | 0.96 (0.80-1.14) | 0.641 |
|  |  | Inverse variance weighted | 15 | 0.93 (0.82-1.06) | 0.260 |
|  |  | Simple mode | 15 | 0.81 (0.60-1.11) | 0.217 |
|  |  | Weighted mode | 15 | 0.97 (0.76-1.25) | 0.820 |
| Family | Coriobacteriaceae | MR Egger | 13 | 0.89 (0.39-2.07) | 0.797 |
|  |  | Weighted median | 13 | 0.94 (0.73-1.21) | 0.638 |
|  |  | Inverse variance weighted | 13 | 0.96 (0.79-1.16) | 0.667 |
|  |  | Simple mode | 13 | 1.01 (0.66-1.56) | 0.959 |
|  |  | Weighted mode | 13 | 0.96 (0.68-1.37) | 0.839 |
| Family | Defluviitaleaceae | MR Egger | 11 | 0.79 (0.49-1.27) | 0.361 |
|  |  | Weighted median | 11 | 1.01 (0.83-1.22) | 0.955 |
|  |  | Inverse variance weighted | 11 | 1.06 (0.92-1.22) | 0.396 |
|  |  | Simple mode | 11 | 0.88 (0.62-1.25) | 0.506 |
|  |  | Weighted mode | 11 | 0.88 (0.63-1.24) | 0.484 |
| Family | Desulfovibrionaceae | MR Egger | 8 | 0.90 (0.18-4.35) | 0.896 |
|  |  | Weighted median | 8 | 1.06 (0.77-1.45) | 0.734 |
|  |  | Inverse variance weighted | 8 | 0.88 (0.65-1.20) | 0.414 |
|  |  | Simple mode | 8 | 1.20 (0.72-2.00) | 0.507 |
|  |  | Weighted mode | 8 | 1.20 (0.76-1.91) | 0.465 |
| Family | Enterobacteriaceae | MR Egger | 7 | 0.31 (0.02-5.87) | 0.473 |
|  |  | Weighted median | 7 | 1.29 (0.86-1.95) | 0.217 |
|  |  | Inverse variance weighted | 7 | 1.09 (0.68-1.77) | 0.711 |
|  |  | Simple mode | 7 | 1.40 (0.62-3.15) | 0.448 |
|  |  | Weighted mode | 7 | 1.41 (0.74-2.68) | 0.336 |
| Family | Erysipelotrichaceae | MR Egger | 13 | 1.90 (0.84-4.31) | 0.153 |
|  |  | Weighted median | 13 | 1.08 (0.83-1.42) | 0.550 |
|  |  | Inverse variance weighted | 13 | 1.02 (0.84-1.23) | 0.876 |
|  |  | Simple mode | 13 | 1.34 (0.82-2.20) | 0.264 |
|  |  | Weighted mode | 13 | 1.36 (0.83-2.23) | 0.249 |
| Family | Eubacteriales Family XII. Incertae Sedis | MR Egger | 8 | 0.89 (0.48-1.65) | 0.718 |
|  |  | Weighted median | 8 | 0.97 (0.86-1.10) | 0.646 |
|  |  | Inverse variance weighted | 8 | 0.98 (0.89-1.07) | 0.613 |
|  |  | Simple mode | 8 | 0.94 (0.76-1.15) | 0.558 |
|  |  | Weighted mode | 8 | 0.92 (0.75-1.13) | 0.466 |
| Family | Eubacteriales Family XIII. Incertae Sedis | MR Egger | 7 | 1.37 (0.55-3.44) | 0.533 |
|  |  | Weighted median | 7 | 0.96 (0.70-1.30) | 0.773 |
|  |  | Inverse variance weighted | 7 | 1.02 (0.79-1.30) | 0.898 |
|  |  | Simple mode | 7 | 0.92 (0.58-1.45) | 0.723 |
|  |  | Weighted mode | 7 | 0.93 (0.60-1.42) | 0.737 |
| Family | Lachnospiraceae | MR Egger | 16 | 1.64 (0.77-3.51) | 0.219 |
|  |  | Weighted median | 16 | 1.12 (0.87-1.44) | 0.368 |
|  |  | Inverse variance weighted | 16 | 1.12 (0.90-1.39) | 0.310 |
|  |  | Simple mode | 16 | 1.19 (0.73-1.94) | 0.491 |
|  |  | Weighted mode | 16 | 1.18 (0.75-1.84) | 0.482 |
| Family | Lactobacillaceae | MR Egger | 8 | 0.95 (0.68-1.31) | 0.754 |
|  |  | Weighted median | 8 | 0.99 (0.84-1.18) | 0.951 |
|  |  | Inverse variance weighted | 8 | 1.03 (0.90-1.17) | 0.705 |
|  |  | Simple mode | 8 | 0.93 (0.72-1.20) | 0.588 |
|  |  | Weighted mode | 8 | 0.98 (0.81-1.19) | 0.858 |
| Family | Methanobacteriaceae | MR Egger | 9 | 1.18 (0.78-1.78) | 0.450 |
|  |  | Weighted median | 9 | 0.96 (0.84-1.11) | 0.616 |
|  |  | Inverse variance weighted | 9 | 0.99 (0.89-1.10) | 0.880 |
|  |  | Simple mode | 9 | 0.88 (0.70-1.12) | 0.338 |
|  |  | Weighted mode | 9 | 0.93 (0.74-1.16) | 0.538 |
| Family | Oxalobacteraceae | MR Egger | 14 | 0.82 (0.52-1.28) | 0.393 |
|  |  | Weighted median | 14 | 0.98 (0.85-1.12) | 0.716 |
|  |  | Inverse variance weighted | 14 | 1.02 (0.91-1.14) | 0.784 |
|  |  | Simple mode | 14 | 0.98 (0.75-1.28) | 0.875 |
|  |  | Weighted mode | 14 | 0.98 (0.74-1.29) | 0.879 |
| Family | Pasteurellaceae | MR Egger | 7 | 0.63 (0.12-3.30) | 0.609 |
|  |  | Weighted median | 7 | 1.24 (0.88-1.75) | 0.219 |
|  |  | Inverse variance weighted | 7 | 1.23 (0.89-1.70) | 0.210 |
|  |  | Simple mode | 7 | 1.50 (0.78-2.88) | 0.268 |
|  |  | Weighted mode | 7 | 1.41 (0.75-2.67) | 0.325 |
| Family | Peptococcaceae | MR Egger | 9 | 0.83 (0.54-1.28) | 0.422 |
|  |  | Weighted median | 9 | 0.94 (0.77-1.14) | 0.506 |
|  |  | Inverse variance weighted | 9 | 0.94 (0.80-1.09) | 0.405 |
|  |  | Simple mode | 9 | 0.95 (0.72-1.25) | 0.719 |
|  |  | Weighted mode | 9 | 0.94 (0.71-1.26) | 0.704 |
| Family | Peptostreptococcaceae | MR Egger | 11 | 1.82 (1.08-3.05) | 0.051 |
|  |  | Weighted median | 11 | 1.16 (0.91-1.46) | 0.224 |
|  |  | Inverse variance weighted | 11 | 1.17 (0.98-1.38) | 0.079 |
|  |  | Simple mode | 11 | 1.08 (0.72-1.62) | 0.722 |
|  |  | Weighted mode | 11 | 1.11 (0.79-1.55) | 0.559 |
| Family | Porphyromonadaceae | MR Egger | 9 | 1.83 (0.38-8.91) | 0.478 |
|  |  | Weighted median | 9 | 1.26 (0.89-1.80) | 0.189 |
|  |  | Inverse variance weighted | 9 | 1.05 (0.74-1.48) | 0.781 |
|  |  | Simple mode | 9 | 1.25 (0.69-2.28) | 0.480 |
|  |  | Weighted mode | 9 | 1.31 (0.78-2.17) | 0.335 |
| Family | Prevotellaceae | MR Egger | 15 | 0.97 (0.46-2.03) | 0.931 |
|  |  | Weighted median | 15 | 0.95 (0.76-1.18) | 0.623 |
|  |  | Inverse variance weighted | 15 | 1.04 (0.85-1.27) | 0.683 |
|  |  | Simple mode | 15 | 0.88 (0.58-1.32) | 0.532 |
|  |  | Weighted mode | 15 | 0.88 (0.63-1.23) | 0.480 |
| Family | Rhodospirillaceae | MR Egger | 15 | 0.82 (0.46-1.45) | 0.497 |
|  |  | Weighted median | 15 | 1.07 (0.92-1.25) | 0.362 |
|  |  | Inverse variance weighted | 15 | 1.02 (0.90-1.17) | 0.741 |
|  |  | Simple mode | 15 | 1.14 (0.87-1.51) | 0.362 |
|  |  | Weighted mode | 15 | 1.07 (0.83-1.36) | 0.613 |
| Family | Rikenellaceae | MR Egger | 16 | 1.46 (0.90-2.36) | 0.150 |
|  |  | Weighted median | 16 | 1.10 (0.88-1.37) | 0.387 |
|  |  | Inverse variance weighted | 16 | 1.11 (0.95-1.30) | 0.185 |
|  |  | Simple mode | 16 | 0.89 (0.58-1.37) | 0.606 |
|  |  | Weighted mode | 16 | 1.13 (0.79-1.63) | 0.505 |
| Family | Oscillospiraceae | MR Egger | 9 | 0.86 (0.56-1.32) | 0.517 |
|  |  | Weighted median | 9 | 1.12 (0.87-1.45) | 0.361 |
|  |  | Inverse variance weighted | 9 | 1.15 (0.95-1.39) | 0.142 |
|  |  | Simple mode | 9 | 1.06 (0.74-1.51) | 0.767 |
|  |  | Weighted mode | 9 | 1.09 (0.79-1.51) | 0.602 |
| Family | Streptococcaceae | MR Egger | 11 | 0.88 (0.40-1.94) | 0.760 |
|  |  | Weighted median | 11 | 0.97 (0.76-1.25) | 0.826 |
|  |  | Inverse variance weighted | 11 | 1.04 (0.86-1.25) | 0.716 |
|  |  | Simple mode | 11 | 0.94 (0.65-1.37) | 0.754 |
|  |  | Weighted mode | 11 | 0.94 (0.67-1.34) | 0.753 |
| Family | unknownfamily | MR Egger | 9 | 1.05 (0.72-1.54) | 0.805 |
|  |  | Weighted median | 9 | 0.98 (0.83-1.16) | 0.824 |
|  |  | Inverse variance weighted | 9 | 0.90 (0.79-1.02) | 0.107 |
|  |  | Simple mode | 9 | 1.00 (0.77-1.29) | 0.972 |
|  |  | Weighted mode | 9 | 1.00 (0.79-1.27) | 0.994 |
| Family | unknownfamily | MR Egger | 12 | 1.04 (0.67-1.61) | 0.864 |
|  |  | Weighted median | 12 | 1.02 (0.84-1.23) | 0.835 |
|  |  | Inverse variance weighted | 12 | 1.02 (0.89-1.18) | 0.757 |
|  |  | Simple mode | 12 | 1.18 (0.88-1.58) | 0.295 |
|  |  | Weighted mode | 12 | 1.06 (0.78-1.45) | 0.710 |
| Family | unknownfamily | MR Egger | 12 | 1.04 (0.59-1.82) | 0.899 |
|  |  | Weighted median | 12 | 0.90 (0.78-1.05) | 0.179 |
|  |  | Inverse variance weighted | 12 | 0.92 (0.80-1.05) | 0.212 |
|  |  | Simple mode | 12 | 0.85 (0.69-1.06) | 0.189 |
|  |  | Weighted mode | 12 | 0.87 (0.71-1.06) | 0.194 |
| Family | Veillonellaceae | MR Egger | 19 | 1.10 (0.85-1.42) | 0.468 |
|  |  | Weighted median | 19 | 1.00 (0.82-1.20) | 0.960 |
|  |  | Inverse variance weighted | 19 | 1.00 (0.88-1.13) | 0.963 |
|  |  | Simple mode | 19 | 0.89 (0.66-1.20) | 0.439 |
|  |  | Weighted mode | 19 | 0.98 (0.77-1.24) | 0.865 |
| Family | Verrucomicrobiaceae | MR Egger | 11 | 1.36 (0.80-2.31) | 0.280 |
|  |  | Weighted median | 11 | 0.91 (0.74-1.12) | 0.377 |
|  |  | Inverse variance weighted | 11 | 0.96 (0.82-1.12) | 0.621 |
|  |  | Simple mode | 11 | 0.84 (0.58-1.21) | 0.374 |
|  |  | Weighted mode | 11 | 0.85 (0.59-1.23) | 0.414 |
| Family | Victivallaceae | MR Egger | 12 | 0.73 (0.49-1.10) | 0.160 |
|  |  | Weighted median | 12 | 1.02 (0.91-1.14) | 0.749 |
|  |  | Inverse variance weighted | 12 | 1.04 (0.95-1.13) | 0.378 |
|  |  | Simple mode | 12 | 1.02 (0.87-1.21) | 0.792 |
|  |  | Weighted mode | 12 | 1.03 (0.87-1.21) | 0.753 |
| Genus | Clostridium (innocuum group) | MR Egger | 7 | 1.12 (0.63- 1.97) | 0.714 |
|  |  | Weighted median | 7 | 0.99 (0.85- 1.15) | 0.867 |
|  |  | Inverse variance weighted | 7 | 0.99 (0.88- 1.11) | 0.867 |
|  |  | Simple mode | 7 | 0.98 (0.80- 1.21) | 0.887 |
|  |  | Weighted mode | 7 | 0.98 (0.80- 1.20) | 0.834 |
| Genus | Eubacterium (brachy group) | MR Egger | 10 | 0.92 (0.61- 1.39) | 0.709 |
|  |  | Weighted median | 10 | 0.98 (0.85- 1.12) | 0.717 |
|  |  | Inverse variance weighted | 10 | 0.98 (0.88- 1.08) | 0.677 |
|  |  | Simple mode | 10 | 0.94 (0.76- 1.16) | 0.590 |
|  |  | Weighted mode | 10 | 0.94 (0.75- 1.18) | 0.628 |
| Genus | Eubacterium (coprostanoligenes group) | MR Egger | 13 | 2.58 (1.25- 5.31) | 0.026 |
|  |  | Weighted median | 13 | 1.04 (0.80- 1.35) | 0.779 |
|  |  | Inverse variance weighted | 13 | 1.10 (0.90- 1.35) | 0.366 |
|  |  | Simple mode | 13 | 0.87 (0.54- 1.39) | 0.563 |
|  |  | Weighted mode | 13 | 0.86 (0.56- 1.34) | 0.520 |
| Genus | Eubacterium (eligens group) | MR Egger | 6 | 1.08 (0.44- 2.66) | 0.881 |
|  |  | Weighted median | 6 | 0.95 (0.71- 1.28) | 0.729 |
|  |  | Inverse variance weighted | 6 | 0.96 (0.75- 1.22) | 0.737 |
|  |  | Simple mode | 6 | 0.88 (0.57- 1.34) | 0.572 |
|  |  | Weighted mode | 6 | 0.92 (0.61- 1.41) | 0.727 |
| Genus | Eubacterium (fissicatena group) | MR Egger | 9 | 1.01 (0.57- 1.76) | 0.984 |
|  |  | Weighted median | 9 | 0.95 (0.83- 1.09) | 0.485 |
|  |  | Inverse variance weighted | 9 | 0.98 (0.88- 1.09) | 0.670 |
|  |  | Simple mode | 9 | 0.93 (0.77- 1.13) | 0.490 |
|  |  | Weighted mode | 9 | 0.94 (0.78- 1.14) | 0.569 |
| Genus | Eubacterium (hallii group) | MR Egger | 15 | 1.17 (0.86- 1.59) | 0.341 |
|  |  | Weighted median | 15 | 0.97 (0.78- 1.19) | 0.746 |
|  |  | Inverse variance weighted | 15 | 1.01 (0.87- 1.17) | 0.871 |
|  |  | Simple mode | 15 | 0.83 (0.58- 1.19) | 0.332 |
|  |  | Weighted mode | 15 | 0.88 (0.65- 1.19) | 0.426 |
| Genus | Eubacterium (nodatum group) | MR Egger | 11 | 1.33 (0.80- 2.21) | 0.302 |
|  |  | Weighted median | 11 | 1.00 (0.87- 1.14) | 0.976 |
|  |  | Inverse variance weighted | 11 | 1.04 (0.93- 1.17) | 0.497 |
|  |  | Simple mode | 11 | 1.00 (0.81- 1.23) | 0.985 |
|  |  | Weighted mode | 11 | 1.00 (0.84- 1.19) | 0.982 |
| Genus | Eubacterium (oxidoreducens group) | MR Egger | 5 | 0.79 (0.26- 2.38) | 0.698 |
|  |  | Weighted median | 5 | 1.14 (0.87- 1.49) | 0.353 |
|  |  | Inverse variance weighted | 5 | 1.08 (0.82- 1.41) | 0.590 |
|  |  | Simple mode | 5 | 1.25 (0.79- 1.99) | 0.393 |
|  |  | Weighted mode | 5 | 1.24 (0.84- 1.84) | 0.336 |
| Genus | Eubacterium (rectale group) | MR Egger | 8 | 2.77 (0.73-10.51) | 0.185 |
|  |  | Weighted median | 8 | 0.93 (0.68- 1.28) | 0.653 |
|  |  | Inverse variance weighted | 8 | 1.14 (0.77- 1.68) | 0.506 |
|  |  | Simple mode | 8 | 0.82 (0.53- 1.27) | 0.412 |
|  |  | Weighted mode | 8 | 0.85 (0.58- 1.25) | 0.441 |
| Genus | Eubacterium (ruminantium group) | MR Egger | 18 | 1.01 (0.72- 1.41) | 0.953 |
|  |  | Weighted median | 18 | 1.05 (0.92- 1.20) | 0.449 |
|  |  | Inverse variance weighted | 18 | 1.04 (0.94- 1.14) | 0.462 |
|  |  | Simple mode | 18 | 1.06 (0.86- 1.31) | 0.576 |
|  |  | Weighted mode | 18 | 1.06 (0.86- 1.31) | 0.576 |
| Genus | Eubacterium (ventriosum group) | MR Egger | 15 | 1.12 (0.56- 2.26) | 0.754 |
|  |  | Weighted median | 15 | 0.99 (0.80- 1.23) | 0.946 |
|  |  | Inverse variance weighted | 15 | 1.07 (0.91- 1.25) | 0.422 |
|  |  | Simple mode | 15 | 0.96 (0.66- 1.39) | 0.838 |
|  |  | Weighted mode | 15 | 0.96 (0.67- 1.37) | 0.819 |
| Genus | Eubacterium (xylanophilum group) | MR Egger | 9 | 0.80 (0.40- 1.60) | 0.550 |
|  |  | Weighted median | 9 | 0.96 (0.75- 1.23) | 0.759 |
|  |  | Inverse variance weighted | 9 | 1.09 (0.87- 1.37) | 0.462 |
|  |  | Simple mode | 9 | 0.92 (0.64- 1.34) | 0.683 |
|  |  | Weighted mode | 9 | 0.90 (0.64- 1.28) | 0.577 |
| Genus | Ruminococcus (gauvreauii group) | MR Egger | 11 | 0.48 (0.23- 0.99) | 0.079 |
|  |  | Weighted median | 11 | 0.88 (0.69- 1.11) | 0.283 |
|  |  | Inverse variance weighted | 11 | 0.95 (0.78- 1.16) | 0.605 |
|  |  | Simple mode | 11 | 0.79 (0.51- 1.22) | 0.315 |
|  |  | Weighted mode | 11 | 0.76 (0.48- 1.20) | 0.267 |
| Genus | Ruminococcus (gnavus group) | MR Egger | 11 | 1.08 (0.61- 1.90) | 0.802 |
|  |  | Weighted median | 11 | 0.95 (0.81- 1.10) | 0.470 |
|  |  | Inverse variance weighted | 11 | 1.00 (0.89- 1.12) | 0.976 |
|  |  | Simple mode | 11 | 0.91 (0.72- 1.16) | 0.475 |
|  |  | Weighted mode | 11 | 0.92 (0.73- 1.17) | 0.514 |
| Genus | Ruminococcus (torques group) | MR Egger | 7 | 1.45 (0.66- 3.20) | 0.398 |
|  |  | Weighted median | 7 | 1.17 (0.84- 1.62) | 0.347 |
|  |  | Inverse variance weighted | 7 | 1.13 (0.87- 1.47) | 0.346 |
|  |  | Simple mode | 7 | 1.27 (0.81- 1.99) | 0.341 |
|  |  | Weighted mode | 7 | 0.99 (0.64- 1.54) | 0.981 |
| Genus | Actinomyces | MR Egger | 7 | 1.01 (0.56- 1.81) | 0.977 |
|  |  | Weighted median | 7 | 0.87 (0.69- 1.10) | 0.251 |
|  |  | Inverse variance weighted | 7 | 0.98 (0.79- 1.22) | 0.846 |
|  |  | Simple mode | 7 | 0.83 (0.58- 1.18) | 0.333 |
|  |  | Weighted mode | 7 | 0.82 (0.62- 1.08) | 0.211 |
| Genus | Adlercreutzia | MR Egger | 8 | 0.91 (0.42- 1.97) | 0.810 |
|  |  | Weighted median | 8 | 0.93 (0.76- 1.14) | 0.487 |
|  |  | Inverse variance weighted | 8 | 0.97 (0.83- 1.15) | 0.742 |
|  |  | Simple mode | 8 | 0.90 (0.66- 1.24) | 0.548 |
|  |  | Weighted mode | 8 | 0.91 (0.68- 1.21) | 0.526 |
| Genus | Akkermansia | MR Egger | 11 | 1.36 (0.80- 2.30) | 0.284 |
|  |  | Weighted median | 11 | 0.91 (0.74- 1.12) | 0.373 |
|  |  | Inverse variance weighted | 11 | 0.96 (0.82- 1.12) | 0.619 |
|  |  | Simple mode | 11 | 0.84 (0.59- 1.20) | 0.363 |
|  |  | Weighted mode | 11 | 0.85 (0.61- 1.20) | 0.376 |
| Genus | Alistipes | MR Egger | 12 | 1.12 (0.42- 2.98) | 0.821 |
|  |  | Weighted median | 12 | 1.13 (0.87- 1.48) | 0.359 |
|  |  | Inverse variance weighted | 12 | 1.14 (0.93- 1.40) | 0.198 |
|  |  | Simple mode | 12 | 1.32 (0.87- 2.00) | 0.219 |
|  |  | Weighted mode | 12 | 1.02 (0.66- 1.57) | 0.928 |
| Genus | Allisonella | MR Egger | 8 | 0.74 (0.32- 1.69) | 0.498 |
|  |  | Weighted median | 8 | 1.04 (0.91- 1.19) | 0.518 |
|  |  | Inverse variance weighted | 8 | 1.03 (0.91- 1.16) | 0.657 |
|  |  | Simple mode | 8 | 1.06 (0.87- 1.28) | 0.601 |
|  |  | Weighted mode | 8 | 1.05 (0.88- 1.27) | 0.597 |
| Genus | Alloprevotella | MR Egger | 6 | 1.08 (0.44- 2.66) | 0.881 |
|  |  | Weighted median | 6 | 0.95 (0.70- 1.29) | 0.736 |
|  |  | Inverse variance weighted | 6 | 0.96 (0.75- 1.22) | 0.737 |
|  |  | Simple mode | 6 | 0.88 (0.59- 1.30) | 0.542 |
|  |  | Weighted mode | 6 | 0.92 (0.63- 1.35) | 0.701 |
| Genus | Anaerofilum | MR Egger | 11 | 0.88 (0.48- 1.63) | 0.696 |
|  |  | Weighted median | 11 | 1.01 (0.87- 1.16) | 0.936 |
|  |  | Inverse variance weighted | 11 | 1.01 (0.90- 1.12) | 0.876 |
|  |  | Simple mode | 11 | 0.99 (0.80- 1.23) | 0.951 |
|  |  | Weighted mode | 11 | 1.00 (0.81- 1.23) | 0.999 |
| Genus | Anaerostipes | MR Egger | 15 | 1.17 (0.86- 1.59) | 0.341 |
|  |  | Weighted median | 15 | 0.97 (0.79- 1.18) | 0.738 |
|  |  | Inverse variance weighted | 15 | 1.01 (0.87- 1.17) | 0.871 |
|  |  | Simple mode | 15 | 0.83 (0.59- 1.18) | 0.324 |
|  |  | Weighted mode | 15 | 0.88 (0.66- 1.18) | 0.416 |
| Genus | Anaerotruncus | MR Egger | 13 | 1.19 (0.60- 2.36) | 0.635 |
|  |  | Weighted median | 13 | 1.10 (0.86- 1.42) | 0.452 |
|  |  | Inverse variance weighted | 13 | 1.07 (0.85- 1.34) | 0.585 |
|  |  | Simple mode | 13 | 1.11 (0.76- 1.63) | 0.588 |
|  |  | Weighted mode | 13 | 1.11 (0.79- 1.56) | 0.565 |
| Genus | Bacteroides | MR Egger | 7 | 0.63 (0.12- 3.30) | 0.609 |
|  |  | Weighted median | 7 | 1.24 (0.87- 1.76) | 0.228 |
|  |  | Inverse variance weighted | 7 | 1.23 (0.89- 1.70) | 0.210 |
|  |  | Simple mode | 7 | 1.50 (0.80- 2.82) | 0.255 |
|  |  | Weighted mode | 7 | 1.41 (0.77- 2.61) | 0.310 |
| Genus | Barnesiella | MR Egger | 12 | 0.85 (0.42- 1.70) | 0.651 |
|  |  | Weighted median | 12 | 0.86 (0.68- 1.08) | 0.194 |
|  |  | Inverse variance weighted | 12 | 0.86 (0.73- 1.02) | 0.087 |
|  |  | Simple mode | 12 | 0.83 (0.58- 1.21) | 0.358 |
|  |  | Weighted mode | 12 | 0.85 (0.59- 1.23) | 0.400 |
| Genus | Bifidobacterium | MR Egger | 12 | 1.30 (0.87- 1.94) | 0.233 |
|  |  | Weighted median | 12 | 0.94 (0.75- 1.17) | 0.582 |
|  |  | Inverse variance weighted | 12 | 1.02 (0.86- 1.20) | 0.853 |
|  |  | Simple mode | 12 | 0.81 (0.54- 1.20) | 0.315 |
|  |  | Weighted mode | 12 | 0.84 (0.49- 1.44) | 0.543 |
| Genus | Bilophila | MR Egger | 13 | 1.13 (0.51- 2.52) | 0.771 |
|  |  | Weighted median | 13 | 1.10 (0.88- 1.38) | 0.379 |
|  |  | Inverse variance weighted | 13 | 1.09 (0.93- 1.29) | 0.277 |
|  |  | Simple mode | 13 | 1.24 (0.82- 1.88) | 0.318 |
|  |  | Weighted mode | 13 | 1.26 (0.84- 1.88) | 0.288 |
| Genus | Blautia | MR Egger | 12 | 0.73 (0.42- 1.28) | 0.295 |
|  |  | Weighted median | 12 | 1.01 (0.76- 1.33) | 0.964 |
|  |  | Inverse variance weighted | 12 | 0.93 (0.75- 1.15) | 0.487 |
|  |  | Simple mode | 12 | 1.02 (0.59- 1.77) | 0.937 |
|  |  | Weighted mode | 12 | 1.09 (0.67- 1.77) | 0.733 |
| Genus | Butyricicoccus | MR Egger | 8 | 0.83 (0.47- 1.46) | 0.546 |
|  |  | Weighted median | 8 | 1.01 (0.75- 1.36) | 0.968 |
|  |  | Inverse variance weighted | 8 | 0.97 (0.74- 1.28) | 0.830 |
|  |  | Simple mode | 8 | 0.84 (0.49- 1.44) | 0.539 |
|  |  | Weighted mode | 8 | 1.03 (0.68- 1.55) | 0.906 |
| Genus | Butyricimonas | MR Egger | 13 | 0.87 (0.45- 1.67) | 0.676 |
|  |  | Weighted median | 13 | 0.85 (0.69- 1.05) | 0.132 |
|  |  | Inverse variance weighted | 13 | 0.99 (0.82- 1.18) | 0.875 |
|  |  | Simple mode | 13 | 0.82 (0.60- 1.10) | 0.214 |
|  |  | Weighted mode | 13 | 0.82 (0.61- 1.10) | 0.210 |
| Genus | Butyrivibrio | MR Egger | 15 | 1.19 (0.82- 1.72) | 0.380 |
|  |  | Weighted median | 15 | 0.97 (0.88- 1.08) | 0.616 |
|  |  | Inverse variance weighted | 15 | 0.99 (0.91- 1.08) | 0.870 |
|  |  | Simple mode | 15 | 0.98 (0.82- 1.17) | 0.794 |
|  |  | Weighted mode | 15 | 0.98 (0.83- 1.15) | 0.779 |
| Genus | Candidatus Soleaferrea | MR Egger | 9 | 0.56 (0.13- 2.44) | 0.466 |
|  |  | Weighted median | 9 | 0.99 (0.82- 1.19) | 0.884 |
|  |  | Inverse variance weighted | 9 | 0.98 (0.85- 1.12) | 0.744 |
|  |  | Simple mode | 9 | 1.07 (0.77- 1.49) | 0.707 |
|  |  | Weighted mode | 9 | 1.07 (0.77- 1.50) | 0.686 |
| Genus | Catenibacterium | MR Egger | 4 | 0.66 (0.05- 9.64) | 0.791 |
|  |  | Weighted median | 4 | 0.97 (0.80- 1.18) | 0.774 |
|  |  | Inverse variance weighted | 4 | 0.95 (0.79- 1.13) | 0.553 |
|  |  | Simple mode | 4 | 0.99 (0.75- 1.30) | 0.932 |
|  |  | Weighted mode | 4 | 0.99 (0.77- 1.27) | 0.946 |
| Genus | Christensenellaceae (R.7 group) | MR Egger | 8 | 1.51 (0.55- 4.10) | 0.454 |
|  |  | Weighted median | 8 | 1.19 (0.84- 1.69) | 0.327 |
|  |  | Inverse variance weighted | 8 | 1.21 (0.93- 1.59) | 0.159 |
|  |  | Simple mode | 8 | 1.26 (0.72- 2.19) | 0.442 |
|  |  | Weighted mode | 8 | 1.15 (0.68- 1.94) | 0.617 |
| Genus | Clostridiumsensustricto 1 | MR Egger | 7 | 1.13 (0.70- 1.83) | 0.637 |
|  |  | Weighted median | 7 | 0.86 (0.66- 1.12) | 0.256 |
|  |  | Inverse variance weighted | 7 | 0.81 (0.67- 0.98) | 0.030 |
|  |  | Simple mode | 7 | 0.85 (0.58- 1.26) | 0.455 |
|  |  | Weighted mode | 7 | 0.86 (0.62- 1.20) | 0.417 |
| Genus | Collinsella | MR Egger | 9 | 0.61 (0.27- 1.34) | 0.256 |
|  |  | Weighted median | 9 | 1.08 (0.80- 1.45) | 0.625 |
|  |  | Inverse variance weighted | 9 | 1.00 (0.81- 1.24) | 0.994 |
|  |  | Simple mode | 9 | 1.23 (0.75- 2.03) | 0.439 |
|  |  | Weighted mode | 9 | 1.23 (0.77- 1.98) | 0.406 |
| Genus | Coprobacter | MR Egger | 10 | 1.92 (1.13- 3.27) | 0.044 |
|  |  | Weighted median | 10 | 1.02 (0.85- 1.23) | 0.826 |
|  |  | Inverse variance weighted | 10 | 1.08 (0.91- 1.28) | 0.395 |
|  |  | Simple mode | 10 | 0.92 (0.64- 1.31) | 0.646 |
|  |  | Weighted mode | 10 | 0.92 (0.65- 1.31) | 0.665 |
| Genus | Coprococcus 1 | MR Egger | 11 | 0.97 (0.63- 1.50) | 0.908 |
|  |  | Weighted median | 11 | 1.06 (0.84- 1.33) | 0.624 |
|  |  | Inverse variance weighted | 11 | 1.10 (0.92- 1.31) | 0.307 |
|  |  | Simple mode | 11 | 0.95 (0.66- 1.35) | 0.775 |
|  |  | Weighted mode | 11 | 0.98 (0.69- 1.39) | 0.922 |
| Genus | Coprococcus 2 | MR Egger | 8 | 2.62 (0.49-13.95) | 0.302 |
|  |  | Weighted median | 8 | 0.95 (0.73- 1.22) | 0.672 |
|  |  | Inverse variance weighted | 8 | 0.95 (0.77- 1.19) | 0.666 |
|  |  | Simple mode | 8 | 0.89 (0.56- 1.42) | 0.642 |
|  |  | Weighted mode | 8 | 0.92 (0.59- 1.43) | 0.712 |
| Genus | Coprococcus 3 | MR Egger | 9 | 0.57 (0.17- 1.87) | 0.386 |
|  |  | Weighted median | 9 | 1.07 (0.82- 1.40) | 0.611 |
|  |  | Inverse variance weighted | 9 | 1.02 (0.83- 1.27) | 0.824 |
|  |  | Simple mode | 9 | 1.07 (0.72- 1.57) | 0.757 |
|  |  | Weighted mode | 9 | 1.06 (0.74- 1.52) | 0.743 |
| Genus | Defluviitaleaceae (UCG011) | MR Egger | 9 | 0.94 (0.54- 1.66) | 0.846 |
|  |  | Weighted median | 9 | 0.96 (0.77- 1.19) | 0.711 |
|  |  | Inverse variance weighted | 9 | 1.00 (0.86- 1.17) | 0.955 |
|  |  | Simple mode | 9 | 0.86 (0.61- 1.23) | 0.441 |
|  |  | Weighted mode | 9 | 0.86 (0.60- 1.24) | 0.446 |
| Genus | Desulfovibrio | MR Egger | 10 | 1.11 (0.67- 1.83) | 0.696 |
|  |  | Weighted median | 10 | 1.12 (0.91- 1.38) | 0.295 |
|  |  | Inverse variance weighted | 10 | 1.06 (0.90- 1.24) | 0.480 |
|  |  | Simple mode | 10 | 1.17 (0.86- 1.58) | 0.346 |
|  |  | Weighted mode | 10 | 1.18 (0.89- 1.57) | 0.288 |
| Genus | Dialister | MR Egger | 11 | 1.15 (0.54- 2.45) | 0.733 |
|  |  | Weighted median | 11 | 1.03 (0.82- 1.29) | 0.809 |
|  |  | Inverse variance weighted | 11 | 1.05 (0.88- 1.25) | 0.603 |
|  |  | Simple mode | 11 | 1.02 (0.74- 1.41) | 0.908 |
|  |  | Weighted mode | 11 | 1.02 (0.74- 1.41) | 0.890 |
| Genus | Dorea | MR Egger | 10 | 1.15 (0.64- 2.07) | 0.645 |
|  |  | Weighted median | 10 | 1.11 (0.84- 1.47) | 0.469 |
|  |  | Inverse variance weighted | 10 | 1.18 (0.95- 1.45) | 0.131 |
|  |  | Simple mode | 10 | 1.13 (0.75- 1.72) | 0.567 |
|  |  | Weighted mode | 10 | 1.12 (0.76- 1.63) | 0.580 |
| Genus | Eggerthella | MR Egger | 10 | 0.96 (0.56- 1.64) | 0.872 |
|  |  | Weighted median | 10 | 1.04 (0.88- 1.22) | 0.639 |
|  |  | Inverse variance weighted | 10 | 1.06 (0.95- 1.20) | 0.290 |
|  |  | Simple mode | 10 | 1.03 (0.81- 1.31) | 0.820 |
|  |  | Weighted mode | 10 | 1.03 (0.81- 1.30) | 0.831 |
| Genus | Eisenbergiella | MR Egger | 11 | 1.22 (0.53- 2.80) | 0.650 |
|  |  | Weighted median | 11 | 1.04 (0.89- 1.22) | 0.584 |
|  |  | Inverse variance weighted | 11 | 1.00 (0.89- 1.11) | 0.938 |
|  |  | Simple mode | 11 | 1.10 (0.86- 1.40) | 0.466 |
|  |  | Weighted mode | 11 | 1.09 (0.85- 1.40) | 0.502 |
| Genus | Enterorhabdus | MR Egger | 6 | 1.20 (0.75- 1.91) | 0.494 |
|  |  | Weighted median | 6 | 1.14 (0.91- 1.43) | 0.248 |
|  |  | Inverse variance weighted | 6 | 1.16 (0.97- 1.39) | 0.093 |
|  |  | Simple mode | 6 | 1.11 (0.82- 1.49) | 0.525 |
|  |  | Weighted mode | 6 | 1.12 (0.85- 1.49) | 0.457 |
| Genus | Erysipelatoclostridium | MR Egger | 15 | 0.59 (0.36- 0.96) | 0.055 |
|  |  | Weighted median | 15 | 0.93 (0.79- 1.09) | 0.366 |
|  |  | Inverse variance weighted | 15 | 0.94 (0.83- 1.06) | 0.304 |
|  |  | Simple mode | 15 | 0.95 (0.72- 1.26) | 0.736 |
|  |  | Weighted mode | 15 | 0.95 (0.72- 1.26) | 0.731 |
| Genus | Erysipelotrichaceae (UCG003) | MR Egger | 16 | 1.01 (0.68- 1.49) | 0.970 |
|  |  | Weighted median | 16 | 0.89 (0.73- 1.08) | 0.245 |
|  |  | Inverse variance weighted | 16 | 0.93 (0.80- 1.07) | 0.287 |
|  |  | Simple mode | 16 | 0.96 (0.69- 1.35) | 0.835 |
|  |  | Weighted mode | 16 | 0.95 (0.70- 1.28) | 0.737 |
| Genus | Escherichia Shigella | MR Egger | 10 | 1.27 (0.73- 2.23) | 0.423 |
|  |  | Weighted median | 10 | 0.98 (0.77- 1.24) | 0.838 |
|  |  | Inverse variance weighted | 10 | 1.01 (0.84- 1.21) | 0.941 |
|  |  | Simple mode | 10 | 0.90 (0.61- 1.32) | 0.593 |
|  |  | Weighted mode | 10 | 0.89 (0.61- 1.31) | 0.577 |
| Genus | Faecalibacterium | MR Egger | 10 | 0.94 (0.66- 1.32) | 0.717 |
|  |  | Weighted median | 10 | 1.06 (0.85- 1.33) | 0.609 |
|  |  | Inverse variance weighted | 10 | 1.07 (0.90- 1.28) | 0.445 |
|  |  | Simple mode | 10 | 1.11 (0.81- 1.54) | 0.529 |
|  |  | Weighted mode | 10 | 1.10 (0.88- 1.39) | 0.424 |
| Genus | Family XIIIAD (3011 group) | MR Egger | 13 | 0.71 (0.28- 1.84) | 0.499 |
|  |  | Weighted median | 13 | 0.92 (0.74- 1.14) | 0.429 |
|  |  | Inverse variance weighted | 13 | 0.99 (0.82- 1.21) | 0.946 |
|  |  | Simple mode | 13 | 0.92 (0.68- 1.25) | 0.614 |
|  |  | Weighted mode | 13 | 0.93 (0.71- 1.22) | 0.613 |
| Genus | FamilyXIIIUCG001 | MR Egger | 8 | 1.26 (0.62- 2.58) | 0.547 |
|  |  | Weighted median | 8 | 0.94 (0.72- 1.22) | 0.628 |
|  |  | Inverse variance weighted | 8 | 0.96 (0.77- 1.21) | 0.751 |
|  |  | Simple mode | 8 | 0.85 (0.58- 1.23) | 0.414 |
|  |  | Weighted mode | 8 | 0.88 (0.60- 1.30) | 0.547 |
| Genus | Flavonifractor | MR Egger | 5 | 0.50 (0.20- 1.21) | 0.221 |
|  |  | Weighted median | 5 | 0.92 (0.69- 1.22) | 0.557 |
|  |  | Inverse variance weighted | 5 | 0.86 (0.69- 1.08) | 0.196 |
|  |  | Simple mode | 5 | 0.92 (0.63- 1.33) | 0.674 |
|  |  | Weighted mode | 5 | 0.92 (0.65- 1.31) | 0.677 |
| Genus | Fusicatenibacter | MR Egger | 18 | 1.31 (0.73- 2.34) | 0.383 |
|  |  | Weighted median | 18 | 0.97 (0.77- 1.22) | 0.792 |
|  |  | Inverse variance weighted | 18 | 1.00 (0.86- 1.17) | 0.988 |
|  |  | Simple mode | 18 | 0.89 (0.61- 1.31) | 0.563 |
|  |  | Weighted mode | 18 | 0.91 (0.62- 1.34) | 0.635 |
| Genus | Gordonibacter | MR Egger | 10 | 1.14 (0.78- 1.69) | 0.517 |
|  |  | Weighted median | 10 | 0.87 (0.76- 0.99) | 0.041 |
|  |  | Inverse variance weighted | 10 | 0.92 (0.83- 1.01) | 0.087 |
|  |  | Simple mode | 10 | 0.86 (0.71- 1.05) | 0.173 |
|  |  | Weighted mode | 10 | 0.86 (0.72- 1.03) | 0.140 |
| Genus | Haemophilus | MR Egger | 9 | 1.06 (0.78- 1.44) | 0.705 |
|  |  | Weighted median | 9 | 1.14 (0.95- 1.36) | 0.172 |
|  |  | Inverse variance weighted | 9 | 1.16 (1.01- 1.33) | 0.032 |
|  |  | Simple mode | 9 | 1.07 (0.83- 1.37) | 0.626 |
|  |  | Weighted mode | 9 | 1.13 (0.89- 1.42) | 0.338 |
| Genus | Holdemanella | MR Egger | 11 | 1.28 (0.91- 1.79) | 0.187 |
|  |  | Weighted median | 11 | 1.00 (0.85- 1.17) | 0.989 |
|  |  | Inverse variance weighted | 11 | 0.98 (0.87- 1.10) | 0.713 |
|  |  | Simple mode | 11 | 1.02 (0.80- 1.31) | 0.871 |
|  |  | Weighted mode | 11 | 1.05 (0.83- 1.32) | 0.693 |
| Genus | Holdemania | MR Egger | 14 | 0.90 (0.60- 1.35) | 0.628 |
|  |  | Weighted median | 14 | 1.00 (0.84- 1.20) | 0.984 |
|  |  | Inverse variance weighted | 14 | 0.96 (0.84- 1.10) | 0.588 |
|  |  | Simple mode | 14 | 1.16 (0.84- 1.60) | 0.386 |
|  |  | Weighted mode | 14 | 1.11 (0.82- 1.50) | 0.507 |
| Genus | Howardella | MR Egger | 9 | 0.82 (0.49- 1.36) | 0.465 |
|  |  | Weighted median | 9 | 0.88 (0.77- 1.01) | 0.073 |
|  |  | Inverse variance weighted | 9 | 0.90 (0.80- 1.01) | 0.075 |
|  |  | Simple mode | 9 | 0.89 (0.70- 1.13) | 0.352 |
|  |  | Weighted mode | 9 | 0.84 (0.67- 1.05) | 0.161 |
| Genus | Hungatella | MR Egger | 5 | 1.17 (0.49- 2.83) | 0.744 |
|  |  | Weighted median | 5 | 0.85 (0.71- 1.03) | 0.098 |
|  |  | Inverse variance weighted | 5 | 0.86 (0.74- 0.99) | 0.040 |
|  |  | Simple mode | 5 | 0.85 (0.68- 1.07) | 0.243 |
|  |  | Weighted mode | 5 | 0.86 (0.68- 1.07) | 0.242 |
| Genus | Intestinibacter | MR Egger | 15 | 1.32 (0.80- 2.19) | 0.303 |
|  |  | Weighted median | 15 | 1.08 (0.88- 1.32) | 0.472 |
|  |  | Inverse variance weighted | 15 | 0.97 (0.83- 1.14) | 0.736 |
|  |  | Simple mode | 15 | 1.11 (0.77- 1.60) | 0.573 |
|  |  | Weighted mode | 15 | 1.15 (0.82- 1.61) | 0.434 |
| Genus | Intestinimonas | MR Egger | 16 | 0.79 (0.52- 1.21) | 0.300 |
|  |  | Weighted median | 16 | 0.91 (0.76- 1.09) | 0.303 |
|  |  | Inverse variance weighted | 16 | 0.88 (0.75- 1.02) | 0.090 |
|  |  | Simple mode | 16 | 0.89 (0.65- 1.22) | 0.483 |
|  |  | Weighted mode | 16 | 0.87 (0.64- 1.17) | 0.370 |
| Genus | Lachnoclostridium | MR Egger | 6 | 1.20 (0.75- 1.91) | 0.494 |
|  |  | Weighted median | 6 | 1.14 (0.91- 1.44) | 0.258 |
|  |  | Inverse variance weighted | 6 | 1.16 (0.97- 1.39) | 0.093 |
|  |  | Simple mode | 6 | 1.11 (0.82- 1.50) | 0.532 |
|  |  | Weighted mode | 6 | 1.12 (0.83- 1.52) | 0.479 |
| Genus | Lachnospira | MR Egger | 6 | 0.04 (0.01- 0.36) | 0.045 |
|  |  | Weighted median | 6 | 0.87 (0.57- 1.31) | 0.500 |
|  |  | Inverse variance weighted | 6 | 0.66 (0.39- 1.13) | 0.129 |
|  |  | Simple mode | 6 | 0.92 (0.54- 1.56) | 0.758 |
|  |  | Weighted mode | 6 | 0.94 (0.57- 1.55) | 0.819 |
| Genus | Lachnospiraceae (FCS020 group) | MR Egger | 16 | 1.01 (0.68- 1.49) | 0.970 |
|  |  | Weighted median | 16 | 0.89 (0.73- 1.09) | 0.263 |
|  |  | Inverse variance weighted | 16 | 0.93 (0.80- 1.07) | 0.287 |
|  |  | Simple mode | 16 | 0.96 (0.70- 1.33) | 0.828 |
|  |  | Weighted mode | 16 | 0.95 (0.69- 1.30) | 0.750 |
| Genus | Lachnospiraceae (NC2004 group) | MR Egger | 9 | 1.17 (0.69- 1.99) | 0.579 |
|  |  | Weighted median | 9 | 1.00 (0.84- 1.19) | 0.997 |
|  |  | Inverse variance weighted | 9 | 0.94 (0.83- 1.07) | 0.380 |
|  |  | Simple mode | 9 | 1.04 (0.80- 1.36) | 0.768 |
|  |  | Weighted mode | 9 | 1.05 (0.83- 1.34) | 0.686 |
| Genus | Lachnospiraceae (ND3007 group) | MR Egger | 3 | 0.04 (0.00-19.87) | 0.494 |
|  |  | Weighted median | 3 | 0.82 (0.52- 1.30) | 0.401 |
|  |  | Inverse variance weighted | 3 | 0.75 (0.52- 1.09) | 0.128 |
|  |  | Simple mode | 3 | 0.84 (0.48- 1.47) | 0.613 |
|  |  | Weighted mode | 3 | 0.84 (0.48- 1.48) | 0.615 |
| Genus | Lachnospiraceae (NK4A136 group) | MR Egger | 15 | 0.98 (0.70- 1.35) | 0.886 |
|  |  | Weighted median | 15 | 1.03 (0.83- 1.28) | 0.776 |
|  |  | Inverse variance weighted | 15 | 1.06 (0.90- 1.24) | 0.497 |
|  |  | Simple mode | 15 | 1.18 (0.85- 1.64) | 0.341 |
|  |  | Weighted mode | 15 | 1.04 (0.83- 1.29) | 0.734 |
| Genus | Lachnospiraceae (UCG001) | MR Egger | 12 | 0.96 (0.55- 1.69) | 0.894 |
|  |  | Weighted median | 12 | 1.08 (0.90- 1.30) | 0.407 |
|  |  | Inverse variance weighted | 12 | 1.04 (0.91- 1.19) | 0.590 |
|  |  | Simple mode | 12 | 1.14 (0.84- 1.55) | 0.421 |
|  |  | Weighted mode | 12 | 1.15 (0.85- 1.57) | 0.391 |
| Genus | Lachnospiraceae (UCG004) | MR Egger | 12 | 1.15 (0.55- 2.39) | 0.720 |
|  |  | Weighted median | 12 | 0.94 (0.74- 1.18) | 0.579 |
|  |  | Inverse variance weighted | 12 | 0.93 (0.78- 1.11) | 0.442 |
|  |  | Simple mode | 12 | 0.92 (0.61- 1.37) | 0.676 |
|  |  | Weighted mode | 12 | 0.91 (0.61- 1.37) | 0.666 |
| Genus | Lachnospiraceae (UCG008) | MR Egger | 10 | 1.10 (0.58- 2.08) | 0.773 |
|  |  | Weighted median | 10 | 0.90 (0.77- 1.05) | 0.165 |
|  |  | Inverse variance weighted | 10 | 0.91 (0.80- 1.03) | 0.137 |
|  |  | Simple mode | 10 | 0.87 (0.68- 1.12) | 0.307 |
|  |  | Weighted mode | 10 | 0.88 (0.70- 1.10) | 0.285 |
| Genus | Lachnospiraceae (UCG010) | MR Egger | 10 | 0.98 (0.56- 1.73) | 0.959 |
|  |  | Weighted median | 10 | 1.02 (0.80- 1.31) | 0.852 |
|  |  | Inverse variance weighted | 10 | 0.98 (0.81- 1.17) | 0.792 |
|  |  | Simple mode | 10 | 1.06 (0.74- 1.53) | 0.755 |
|  |  | Weighted mode | 10 | 1.06 (0.74- 1.53) | 0.743 |
| Genus | Lactobacillus | MR Egger | 8 | 1.00 (0.72- 1.39) | 0.988 |
|  |  | Weighted median | 8 | 1.05 (0.89- 1.25) | 0.540 |
|  |  | Inverse variance weighted | 8 | 1.07 (0.94- 1.21) | 0.326 |
|  |  | Simple mode | 8 | 1.05 (0.85- 1.31) | 0.661 |
|  |  | Weighted mode | 8 | 1.05 (0.85- 1.29) | 0.649 |
| Genus | Lactococcus | MR Egger | 9 | 0.81 (0.42- 1.56) | 0.546 |
|  |  | Weighted median | 9 | 0.90 (0.77- 1.05) | 0.166 |
|  |  | Inverse variance weighted | 9 | 0.94 (0.81- 1.08) | 0.347 |
|  |  | Simple mode | 9 | 0.82 (0.62- 1.10) | 0.226 |
|  |  | Weighted mode | 9 | 0.89 (0.69- 1.15) | 0.398 |
| Genus | Marvinbryantia | MR Egger | 10 | 1.45 (0.72- 2.88) | 0.326 |
|  |  | Weighted median | 10 | 1.00 (0.79- 1.27) | 0.983 |
|  |  | Inverse variance weighted | 10 | 1.00 (0.84- 1.20) | 0.977 |
|  |  | Simple mode | 10 | 1.04 (0.74- 1.45) | 0.833 |
|  |  | Weighted mode | 10 | 1.03 (0.74- 1.43) | 0.884 |
| Genus | Methanobrevibacter | MR Egger | 6 | 1.25 (0.66- 2.39) | 0.530 |
|  |  | Weighted median | 6 | 1.06 (0.88- 1.28) | 0.564 |
|  |  | Inverse variance weighted | 6 | 0.98 (0.83- 1.15) | 0.791 |
|  |  | Simple mode | 6 | 1.05 (0.80- 1.37) | 0.756 |
|  |  | Weighted mode | 6 | 1.05 (0.85- 1.31) | 0.667 |
| Genus | Odoribacter | MR Egger | 7 | 1.04 (0.37- 2.91) | 0.942 |
|  |  | Weighted median | 7 | 0.97 (0.71- 1.33) | 0.837 |
|  |  | Inverse variance weighted | 7 | 1.16 (0.86- 1.56) | 0.338 |
|  |  | Simple mode | 7 | 0.87 (0.56- 1.36) | 0.571 |
|  |  | Weighted mode | 7 | 0.88 (0.59- 1.30) | 0.538 |
| Genus | Olsenella | MR Egger | 10 | 1.33 (0.95- 1.87) | 0.137 |
|  |  | Weighted median | 10 | 1.03 (0.91- 1.18) | 0.624 |
|  |  | Inverse variance weighted | 10 | 0.99 (0.88- 1.11) | 0.818 |
|  |  | Simple mode | 10 | 0.95 (0.79- 1.14) | 0.567 |
|  |  | Weighted mode | 10 | 1.10 (0.94- 1.29) | 0.253 |
| Genus | Oscillibacter | MR Egger | 13 | 1.36 (0.84- 2.20) | 0.235 |
|  |  | Weighted median | 13 | 1.12 (0.94- 1.33) | 0.198 |
|  |  | Inverse variance weighted | 13 | 0.98 (0.86- 1.12) | 0.812 |
|  |  | Simple mode | 13 | 1.15 (0.84- 1.56) | 0.403 |
|  |  | Weighted mode | 13 | 1.15 (0.86- 1.52) | 0.366 |
| Genus | Oscillospira | MR Egger | 8 | 0.64 (0.29- 1.41) | 0.311 |
|  |  | Weighted median | 8 | 1.00 (0.78- 1.27) | 0.971 |
|  |  | Inverse variance weighted | 8 | 0.91 (0.75- 1.09) | 0.312 |
|  |  | Simple mode | 8 | 1.05 (0.71- 1.57) | 0.801 |
|  |  | Weighted mode | 8 | 1.05 (0.72- 1.52) | 0.825 |
| Genus | Oxalobacter | MR Egger | 11 | 0.78 (0.50- 1.24) | 0.319 |
|  |  | Weighted median | 11 | 0.97 (0.85- 1.11) | 0.667 |
|  |  | Inverse variance weighted | 11 | 0.99 (0.90- 1.09) | 0.811 |
|  |  | Simple mode | 11 | 0.96 (0.76- 1.21) | 0.729 |
|  |  | Weighted mode | 11 | 0.96 (0.77- 1.20) | 0.730 |
| Genus | Parabacteroides | MR Egger | 5 | 0.11 (0.02- 0.62) | 0.087 |
|  |  | Weighted median | 5 | 0.98 (0.67- 1.44) | 0.916 |
|  |  | Inverse variance weighted | 5 | 1.09 (0.72- 1.63) | 0.688 |
|  |  | Simple mode | 5 | 0.93 (0.50- 1.73) | 0.828 |
|  |  | Weighted mode | 5 | 0.92 (0.55- 1.54) | 0.774 |
| Genus | Paraprevotella | MR Egger | 13 | 0.73 (0.47- 1.13) | 0.185 |
|  |  | Weighted median | 13 | 0.97 (0.83- 1.13) | 0.680 |
|  |  | Inverse variance weighted | 13 | 1.00 (0.88- 1.13) | 0.984 |
|  |  | Simple mode | 13 | 1.04 (0.78- 1.39) | 0.801 |
|  |  | Weighted mode | 13 | 1.03 (0.79- 1.35) | 0.819 |
| Genus | Parasutterella | MR Egger | 14 | 1.22 (0.86- 1.75) | 0.293 |
|  |  | Weighted median | 14 | 1.07 (0.90- 1.28) | 0.451 |
|  |  | Inverse variance weighted | 14 | 1.07 (0.94- 1.22) | 0.276 |
|  |  | Simple mode | 14 | 1.05 (0.79- 1.40) | 0.737 |
|  |  | Weighted mode | 14 | 1.08 (0.83- 1.41) | 0.558 |
| Genus | Peptococcus | MR Egger | 12 | 0.92 (0.62- 1.37) | 0.696 |
|  |  | Weighted median | 12 | 1.07 (0.93- 1.24) | 0.347 |
|  |  | Inverse variance weighted | 12 | 1.03 (0.93- 1.15) | 0.515 |
|  |  | Simple mode | 12 | 1.12 (0.88- 1.42) | 0.392 |
|  |  | Weighted mode | 12 | 1.10 (0.87- 1.38) | 0.437 |
| Genus | Phascolarctobacterium | MR Egger | 8 | 1.02 (0.39- 2.66) | 0.963 |
|  |  | Weighted median | 8 | 0.95 (0.74- 1.22) | 0.675 |
|  |  | Inverse variance weighted | 8 | 0.99 (0.82- 1.20) | 0.930 |
|  |  | Simple mode | 8 | 0.93 (0.63- 1.38) | 0.724 |
|  |  | Weighted mode | 8 | 0.94 (0.66- 1.32) | 0.715 |
| Genus | Prevotella 7 | MR Egger | 11 | 1.15 (0.66-2.01) | 0.630 |
|  |  | Weighted median | 11 | 0.99 (0.88-1.12) | 0.908 |
|  |  | Inverse variance weighted | 11 | 0.99 (0.90-1.08) | 0.774 |
|  |  | Simple mode | 11 | 0.98 (0.82-1.19) | 0.872 |
|  |  | Weighted mode | 11 | 0.98 (0.83-1.17) | 0.864 |
| Genus | Prevotella 9 | MR Egger | 15 | 0.86 (0.61-1.22) | 0.408 |
|  |  | Weighted median | 15 | 0.93 (0.79-1.09) | 0.391 |
|  |  | Inverse variance weighted | 15 | 0.93 (0.83-1.05) | 0.263 |
|  |  | Simple mode | 15 | 0.94 (0.71-1.24) | 0.656 |
|  |  | Weighted mode | 15 | 0.92 (0.71-1.19) | 0.538 |
| Genus | Rikenellaceae | MR Egger | 11 | 0.62 (0.35-1.09) | 0.131 |
|  |  | Weighted median | 11 | 0.96 (0.84-1.10) | 0.528 |
|  |  | Inverse variance weighted | 11 | 0.96 (0.87-1.06) | 0.401 |
|  |  | Simple mode | 11 | 0.89 (0.69-1.14) | 0.373 |
|  |  | Weighted mode | 11 | 0.89 (0.70-1.14) | 0.378 |
| Genus | Romboutsia | MR Egger | 13 | 1.08 (0.44-2.66) | 0.873 |
|  |  | Weighted median | 13 | 1.06 (0.81-1.39) | 0.654 |
|  |  | Inverse variance weighted | 13 | 0.94 (0.69-1.26) | 0.666 |
|  |  | Simple mode | 13 | 1.29 (0.72-2.30) | 0.416 |
|  |  | Weighted mode | 13 | 1.01 (0.66-1.56) | 0.963 |
| Genus | Roseburia | MR Egger | 14 | 1.24 (0.72-2.13) | 0.448 |
|  |  | Weighted median | 14 | 1.05 (0.83-1.32) | 0.703 |
|  |  | Inverse variance weighted | 14 | 1.08 (0.91-1.29) | 0.379 |
|  |  | Simple mode | 14 | 0.98 (0.66-1.46) | 0.926 |
|  |  | Weighted mode | 14 | 0.99 (0.68-1.45) | 0.969 |
| Genus | Ruminiclostridium 5 | MR Egger | 11 | 0.80 (0.22-2.89) | 0.744 |
|  |  | Weighted median | 11 | 1.00 (0.74-1.35) | 0.990 |
|  |  | Inverse variance weighted | 11 | 1.11 (0.83-1.49) | 0.481 |
|  |  | Simple mode | 11 | 0.98 (0.59-1.63) | 0.927 |
|  |  | Weighted mode | 11 | 0.98 (0.59-1.61) | 0.926 |
| Genus | Ruminiclostridium 6 | MR Egger | 14 | 0.85 (0.55-1.29) | 0.451 |
|  |  | Weighted median | 14 | 1.02 (0.82-1.27) | 0.879 |
|  |  | Inverse variance weighted | 14 | 0.98 (0.82-1.16) | 0.802 |
|  |  | Simple mode | 14 | 1.10 (0.74-1.63) | 0.653 |
|  |  | Weighted mode | 14 | 1.19 (0.87-1.62) | 0.299 |
| Genus | Ruminiclostridium 9 | MR Egger | 7 | 0.70 (0.22-2.16) | 0.557 |
|  |  | Weighted median | 7 | 0.98 (0.72-1.33) | 0.887 |
|  |  | Inverse variance weighted | 7 | 0.95 (0.74-1.22) | 0.685 |
|  |  | Simple mode | 7 | 0.99 (0.60-1.62) | 0.968 |
|  |  | Weighted mode | 7 | 0.99 (0.65-1.52) | 0.972 |
| Genus | Ruminococcaceae (NK4A214 group) | MR Egger | 13 | 0.73 (0.42-1.27) | 0.283 |
|  |  | Weighted median | 13 | 0.99 (0.77-1.26) | 0.922 |
|  |  | Inverse variance weighted | 13 | 0.98 (0.83-1.17) | 0.856 |
|  |  | Simple mode | 13 | 0.87 (0.58-1.32) | 0.533 |
|  |  | Weighted mode | 13 | 0.91 (0.63-1.32) | 0.628 |
| Genus | Ruminococcaceae (UCG002) | MR Egger | 19 | 1.02 (0.67-1.56) | 0.915 |
|  |  | Weighted median | 19 | 1.05 (0.85-1.30) | 0.659 |
|  |  | Inverse variance weighted | 19 | 0.95 (0.81-1.11) | 0.504 |
|  |  | Simple mode | 19 | 1.00 (0.65-1.53) | 0.999 |
|  |  | Weighted mode | 19 | 1.06 (0.73-1.52) | 0.764 |
| Genus | Ruminococcaceae (UCG003) | MR Egger | 12 | 0.98 (0.58-1.69) | 0.957 |
|  |  | Weighted median | 12 | 1.08 (0.87-1.34) | 0.469 |
|  |  | Inverse variance weighted | 12 | 1.04 (0.88-1.22) | 0.669 |
|  |  | Simple mode | 12 | 1.12 (0.77-1.62) | 0.569 |
|  |  | Weighted mode | 12 | 1.07 (0.74-1.56) | 0.712 |
| Genus | Ruminococcaceae (UCG004) | MR Egger | 11 | 1.37 (0.44-4.31) | 0.602 |
|  |  | Weighted median | 11 | 1.14 (0.92-1.41) | 0.232 |
|  |  | Inverse variance weighted | 11 | 1.03 (0.85-1.26) | 0.752 |
|  |  | Simple mode | 11 | 1.26 (0.87-1.82) | 0.254 |
|  |  | Weighted mode | 11 | 1.20 (0.85-1.68) | 0.322 |
| Genus | Ruminococcaceae (UCG005) | MR Egger | 14 | 0.91 (0.61-1.37) | 0.672 |
|  |  | Weighted median | 14 | 1.01 (0.83-1.24) | 0.901 |
|  |  | Inverse variance weighted | 14 | 1.03 (0.89-1.20) | 0.659 |
|  |  | Simple mode | 14 | 1.01 (0.75-1.36) | 0.967 |
|  |  | Weighted mode | 14 | 1.02 (0.79-1.32) | 0.864 |
| Genus | Ruminococcaceae (UCG009) | MR Egger | 12 | 1.27 (0.78-2.06) | 0.360 |
|  |  | Weighted median | 12 | 0.92 (0.78-1.08) | 0.314 |
|  |  | Inverse variance weighted | 12 | 0.89 (0.79-1.01) | 0.066 |
|  |  | Simple mode | 12 | 0.92 (0.70-1.21) | 0.584 |
|  |  | Weighted mode | 12 | 0.95 (0.73-1.23) | 0.706 |
| Genus | Ruminococcaceae (UCG010) | MR Egger | 6 | 1.15 (0.49-2.72) | 0.759 |
|  |  | Weighted median | 6 | 1.27 (0.94-1.72) | 0.115 |
|  |  | Inverse variance weighted | 6 | 1.38 (1.04-1.84) | 0.028 |
|  |  | Simple mode | 6 | 1.31 (0.85-2.02) | 0.280 |
|  |  | Weighted mode | 6 | 1.28 (0.92-1.79) | 0.203 |
| Genus | Ruminococcaceae (UCG011) | MR Egger | 8 | 1.15 (0.64-2.06) | 0.666 |
|  |  | Weighted median | 8 | 1.04 (0.90-1.19) | 0.627 |
|  |  | Inverse variance weighted | 8 | 0.92 (0.82-1.03) | 0.165 |
|  |  | Simple mode | 8 | 1.03 (0.82-1.30) | 0.802 |
|  |  | Weighted mode | 8 | 1.04 (0.85-1.26) | 0.738 |
| Genus | Ruminococcaceae (UCG013) | MR Egger | 11 | 0.94 (0.50-1.79) | 0.862 |
|  |  | Weighted median | 11 | 0.87 (0.68-1.12) | 0.278 |
|  |  | Inverse variance weighted | 11 | 0.92 (0.74-1.14) | 0.437 |
|  |  | Simple mode | 11 | 0.83 (0.51-1.33) | 0.452 |
|  |  | Weighted mode | 11 | 0.80 (0.50-1.27) | 0.369 |
| Genus | Ruminococcaceae (UCG014) | MR Egger | 11 | 0.75 (0.51-1.09) | 0.166 |
|  |  | Weighted median | 11 | 0.86 (0.69-1.08) | 0.196 |
|  |  | Inverse variance weighted | 11 | 0.90 (0.77-1.05) | 0.192 |
|  |  | Simple mode | 11 | 0.87 (0.64-1.16) | 0.363 |
|  |  | Weighted mode | 11 | 0.86 (0.67-1.10) | 0.254 |
| Genus | Ruminococcus 1 | MR Egger | 10 | 0.83 (0.45-1.55) | 0.580 |
|  |  | Weighted median | 10 | 0.96 (0.76-1.22) | 0.745 |
|  |  | Inverse variance weighted | 10 | 0.90 (0.73-1.13) | 0.365 |
|  |  | Simple mode | 10 | 1.03 (0.72-1.47) | 0.864 |
|  |  | Weighted mode | 10 | 1.08 (0.74-1.57) | 0.700 |
| Genus | Ruminococcus 2 | MR Egger | 15 | 1.01 (0.69-1.49) | 0.945 |
|  |  | Weighted median | 15 | 1.05 (0.87-1.28) | 0.590 |
|  |  | Inverse variance weighted | 15 | 1.01 (0.86-1.17) | 0.938 |
|  |  | Simple mode | 15 | 0.96 (0.71-1.29) | 0.794 |
|  |  | Weighted mode | 15 | 0.98 (0.77-1.26) | 0.902 |
| Genus | Sellimonas | MR Egger | 13 | 1.08 (0.44-2.66) | 0.873 |
|  |  | Weighted median | 13 | 1.06 (0.81-1.39) | 0.653 |
|  |  | Inverse variance weighted | 13 | 0.94 (0.69-1.26) | 0.666 |
|  |  | Simple mode | 13 | 1.29 (0.75-2.19) | 0.375 |
|  |  | Weighted mode | 13 | 1.01 (0.64-1.60) | 0.965 |
| Genus | Senegalimassilia | MR Egger | 5 | 0.97 (0.45-2.11) | 0.951 |
|  |  | Weighted median | 5 | 0.96 (0.74-1.26) | 0.792 |
|  |  | Inverse variance weighted | 5 | 1.02 (0.83-1.24) | 0.856 |
|  |  | Simple mode | 5 | 0.93 (0.65-1.32) | 0.698 |
|  |  | Weighted mode | 5 | 0.94 (0.67-1.32) | 0.733 |
| Genus | Slackia | MR Egger | 11 | 0.80 (0.22-2.89) | 0.744 |
|  |  | Weighted median | 11 | 1.00 (0.74-1.35) | 0.990 |
|  |  | Inverse variance weighted | 11 | 1.11 (0.83-1.49) | 0.481 |
|  |  | Simple mode | 11 | 0.98 (0.59-1.62) | 0.926 |
|  |  | Weighted mode | 11 | 0.98 (0.59-1.62) | 0.927 |
| Genus | Streptococcus | MR Egger | 12 | 1.04 (0.52-2.05) | 0.920 |
|  |  | Weighted median | 12 | 0.96 (0.75-1.23) | 0.743 |
|  |  | Inverse variance weighted | 12 | 0.99 (0.82-1.19) | 0.903 |
|  |  | Simple mode | 12 | 0.97 (0.65-1.45) | 0.883 |
|  |  | Weighted mode | 12 | 0.96 (0.68-1.37) | 0.838 |
| Genus | Subdoligranulum | MR Egger | 11 | 0.98 (0.62-1.55) | 0.936 |
|  |  | Weighted median | 11 | 1.18 (0.93-1.51) | 0.179 |
|  |  | Inverse variance weighted | 11 | 1.27 (1.06-1.52) | 0.009 |
|  |  | Simple mode | 11 | 1.18 (0.85-1.63) | 0.348 |
|  |  | Weighted mode | 11 | 1.18 (0.87-1.60) | 0.317 |
| Genus | Sutterella | MR Egger | 12 | 1.82 (0.90-3.70) | 0.127 |
|  |  | Weighted median | 12 | 1.08 (0.86-1.34) | 0.522 |
|  |  | Inverse variance weighted | 12 | 1.10 (0.94-1.30) | 0.245 |
|  |  | Simple mode | 12 | 1.06 (0.69-1.63) | 0.800 |
|  |  | Weighted mode | 12 | 1.08 (0.71-1.63) | 0.740 |
| Genus | Terrisporobacter | MR Egger | 5 | 1.06 (0.45-2.51) | 0.899 |
|  |  | Weighted median | 5 | 1.05 (0.82-1.34) | 0.688 |
|  |  | Inverse variance weighted | 5 | 1.10 (0.86-1.41) | 0.438 |
|  |  | Simple mode | 5 | 1.01 (0.76-1.35) | 0.932 |
|  |  | Weighted mode | 5 | 1.00 (0.76-1.32) | 0.986 |
| Genus | Turicibacter | MR Egger | 9 | 1.15 (0.62-2.14) | 0.676 |
|  |  | Weighted median | 9 | 0.97 (0.79-1.19) | 0.753 |
|  |  | Inverse variance weighted | 9 | 0.95 (0.82-1.11) | 0.527 |
|  |  | Simple mode | 9 | 0.96 (0.70-1.32) | 0.828 |
|  |  | Weighted mode | 9 | 0.99 (0.72-1.35) | 0.949 |
| Genus | Tyzzerella 3 | MR Egger | 13 | 0.68 (0.38-1.22) | 0.219 |
|  |  | Weighted median | 13 | 1.01 (0.88-1.16) | 0.905 |
|  |  | Inverse variance weighted | 13 | 1.01 (0.91-1.12) | 0.861 |
|  |  | Simple mode | 13 | 0.98 (0.79-1.21) | 0.828 |
|  |  | Weighted mode | 13 | 0.98 (0.80-1.21) | 0.871 |
| Genus | Unknown genus(826) | MR Egger | 15 | 1.16 (0.82-1.64) | 0.414 |
|  |  | Weighted median | 15 | 0.96 (0.80-1.15) | 0.653 |
|  |  | Inverse variance weighted | 15 | 0.93 (0.82-1.06) | 0.260 |
|  |  | Simple mode | 15 | 0.81 (0.60-1.10) | 0.201 |
|  |  | Weighted mode | 15 | 0.97 (0.76-1.24) | 0.814 |
| Genus | Unknown genus (959) | MR Egger | 9 | 1.05 (0.72-1.54) | 0.805 |
|  |  | Weighted median | 9 | 0.98 (0.83-1.16) | 0.822 |
|  |  | Inverse variance weighted | 9 | 0.90 (0.79-1.02) | 0.107 |
|  |  | Simple mode | 9 | 1.00 (0.78-1.27) | 0.971 |
|  |  | Weighted mode | 9 | 1.00 (0.80-1.25) | 0.994 |
| Genus | Unknown genus (1868) | MR Egger | 12 | 1.04 (0.67-1.61) | 0.864 |
|  |  | Weighted median | 12 | 1.02 (0.84-1.24) | 0.841 |
|  |  | Inverse variance weighted | 12 | 1.02 (0.89-1.18) | 0.757 |
|  |  | Simple mode | 12 | 1.18 (0.86-1.61) | 0.321 |
|  |  | Weighted mode | 12 | 1.06 (0.81-1.40) | 0.671 |
| Genus | Unknown genus (2001) | MR Egger | 8 | 1.21 (0.67-2.17) | 0.556 |
|  |  | Weighted median | 8 | 1.08 (0.88-1.32) | 0.455 |
|  |  | Inverse variance weighted | 8 | 0.95 (0.82-1.10) | 0.527 |
|  |  | Simple mode | 8 | 1.09 (0.79-1.50) | 0.624 |
|  |  | Weighted mode | 8 | 1.09 (0.82-1.46) | 0.564 |
| Genus | Unknown genus (2041) | MR Egger | 12 | 1.04 (0.59-1.82) | 0.899 |
|  |  | Weighted median | 12 | 0.90 (0.79-1.04) | 0.166 |
|  |  | Inverse variance weighted | 12 | 0.92 (0.80-1.05) | 0.212 |
|  |  | Simple mode | 12 | 0.85 (0.69-1.07) | 0.193 |
|  |  | Weighted mode | 12 | 0.87 (0.71-1.06) | 0.198 |
| Genus | Unknown genus(2071) | MR Egger | 10 | 0.77 (0.39-1.53) | 0.477 |
|  |  | Weighted median | 10 | 0.95 (0.75-1.19) | 0.640 |
|  |  | Inverse variance weighted | 10 | 0.96 (0.79-1.18) | 0.704 |
|  |  | Simple mode | 10 | 0.80 (0.51-1.28) | 0.377 |
|  |  | Weighted mode | 10 | 0.78 (0.54-1.15) | 0.244 |
| Genus | Unknown genus (2755) | MR Egger | 9 | 1.17 (0.73-1.87) | 0.543 |
|  |  | Weighted median | 9 | 1.00 (0.80-1.26) | 0.984 |
|  |  | Inverse variance weighted | 9 | 0.97 (0.82-1.15) | 0.711 |
|  |  | Simple mode | 9 | 1.13 (0.81-1.58) | 0.480 |
|  |  | Weighted mode | 9 | 1.06 (0.84-1.33) | 0.658 |
| Genus | Unknown genus(1000000073) | MR Egger | 12 | 0.85 (0.53-1.35) | 0.503 |
|  |  | Weighted median | 12 | 1.06 (0.89-1.27) | 0.521 |
|  |  | Inverse variance weighted | 12 | 0.95 (0.83-1.09) | 0.475 |
|  |  | Simple mode | 12 | 1.16 (0.82-1.64) | 0.426 |
|  |  | Weighted mode | 12 | 1.16 (0.84-1.60) | 0.389 |
| Genus | Unknown genus (1000001215) | MR Egger | 15 | 0.71 (0.35-1.47) | 0.374 |
|  |  | Weighted median | 15 | 1.07 (0.87-1.31) | 0.514 |
|  |  | Inverse variance weighted | 15 | 0.98 (0.85-1.13) | 0.775 |
|  |  | Simple mode | 15 | 1.14 (0.80-1.63) | 0.483 |
|  |  | Weighted mode | 15 | 1.14 (0.79-1.64) | 0.487 |
| Genus | Unknown genus(1000005472) | MR Egger | 13 | 0.83 (0.53-1.29) | 0.420 |
|  |  | Weighted median | 13 | 1.08 (0.92-1.26) | 0.369 |
|  |  | Inverse variance weighted | 13 | 1.10 (0.98-1.23) | 0.117 |
|  |  | Simple mode | 13 | 1.09 (0.83-1.43) | 0.551 |
|  |  | Weighted mode | 13 | 1.09 (0.84-1.40) | 0.528 |
| Genus | Unknown genus (1000005479) | MR Egger | 13 | 0.81 (0.54-1.23) | 0.349 |
|  |  | Weighted median | 13 | 0.95 (0.78-1.15) | 0.603 |
|  |  | Inverse variance weighted | 13 | 0.96 (0.83-1.11) | 0.581 |
|  |  | Simple mode | 13 | 0.91 (0.65-1.26) | 0.569 |
|  |  | Weighted mode | 13 | 0.95 (0.71-1.29) | 0.759 |
| Genus | Unknown genus (1000006162) | MR Egger | 11 | 0.71 (0.42-1.20) | 0.231 |
|  |  | Weighted median | 11 | 0.98 (0.85-1.14) | 0.826 |
|  |  | Inverse variance weighted | 11 | 0.96 (0.87-1.06) | 0.436 |
|  |  | Simple mode | 11 | 1.00 (0.81-1.25) | 0.970 |
|  |  | Weighted mode | 11 | 1.00 (0.81-1.23) | 0.974 |
| Genus | Veillonella | MR Egger | 6 | 1.31 (0.26-6.73) | 0.760 |
|  |  | Weighted median | 6 | 1.09 (0.84-1.41) | 0.523 |
|  |  | Inverse variance weighted | 6 | 0.97 (0.80-1.18) | 0.782 |
|  |  | Simple mode | 6 | 1.14 (0.76-1.71) | 0.564 |
|  |  | Weighted mode | 6 | 1.14 (0.76-1.71) | 0.551 |
| Genus | Victivallis | MR Egger | 10 | 1.19 (0.41-3.43) | 0.760 |
|  |  | Weighted median | 10 | 1.00 (0.87-1.15) | 0.967 |
|  |  | Inverse variance weighted | 10 | 1.05 (0.92-1.19) | 0.501 |
|  |  | Simple mode | 10 | 0.94 (0.74-1.19) | 0.611 |
|  |  | Weighted mode | 10 | 0.93 (0.76-1.15) | 0.520 |

**Table S3 Reverse MR results of causal links between gut microbiome and kidney stones risk.**

| **Group** | **Gut microbiota** | **MR method** | **No.SNP** | **OR (95% CI)** | **P-value** |
| --- | --- | --- | --- | --- | --- |
| Genus | Oxalobacter | Inverse variance weighted | 4 | 0.98 (0.81 - 1.19) | 0.863 |
|  |  | MR Egger | 4 | 1.11 (0.43 - 2.86) | 0.851 |
|  |  | Weighted median | 4 | 0.96 (0.78 - 1.19) | 0.711 |
|  |  | Simple mode | 4 | 0.95 (0.72 - 1.25) | 0.723 |
|  |  | Weighted mode | 4 | 0.94 (0.72 - 1.25) | 0.714 |
| Order | Actinomycetales | Inverse variance weighted | 4 | 0.93 (0.80 - 1.08) | 0.320 |
|  |  | MR Egger | 4 | 0.95 (0.46 - 1.97) | 0.898 |
|  |  | Weighted median | 4 | 0.93 (0.79 - 1.11 | 0.432 |
|  |  | Simple mode | 4 | 0.94 (0.75 - 1.18) | 0.636 |
|  |  | Weighted mode | 4 | 0.94 (0.76 - 1.15) | 0.585 |
| Family | Actinomycetaceae | Inverse variance weighted | 4 | 0.93 (0.80 - 1.08) | 0.326 |
|  |  | MR Egger | 4 | 0.95 (0.46 - 1.98) | 0.907 |
|  |  | Weighted median | 4 | 0.94 (0.79 - 1.10) | 0.425 |
|  |  | Simple mode | 4 | 0.94 (0.75 - 1.18) | 0.645 |
|  |  | Weighted mode | 4 | 0.94 (0.76 - 1.16) | 0.603 |
| Family | Clostridiaceae 1 | Inverse variance weighted | 4 | 0.94 (0.84 - 1.04) | 0.236 |
|  |  | MR Egger | 4 | 0.99 (0.58 - 1.69) | 0.962 |
|  |  | Weighted median | 4 | 0.93 (0.82 - 1.06) | 0.280 |
|  |  | Simple mode | 4 | 0.93 (0.79 - 1.09) | 0.448 |
|  |  | Weighted mode | 4 | 0.92 (0.78 - 1.08) | 0.380 |
| Genus | Clostridiumsensustricto 1 | Inverse variance weighted | 4 | 0.95 (0.85 - 1.06) | 0.362 |
|  |  | MR Egger | 4 | 0.99 (0.58 - 1.70) | 0.973 |
|  |  | Weighted median | 4 | 0.95 (0.84 - 1.08) | 0.421 |
|  |  | Simple mode | 4 | 0.95 (0.80 - 1.12) | 0.576 |
|  |  | Weighted mode | 4 | 0.94 (0.80 - 1.09) | 0.474 |
| Genus | Haemophilus | Inverse variance weighted | 4 | 1.02 (0.89 - 1.17) | 0.740 |
|  |  | MR Egger | 4 | 0.78 (0.40 - 1.51) | 0.534 |
|  |  | Weighted median | 4 | 1.02 (0.87 - 1.19) | 0.821 |
|  |  | Simple mode | 4 | 1.01 (0.81 - 1.27) | 0.905 |
|  |  | Weighted mode | 4 | 1.00 (0.82 - 1.23) | 0.987 |
| Genus | Hungatella | Inverse variance weighted | 4 | 0.94 (0.77 - 1.15) | 0.571 |
|  |  | MR Egger | 4 | 0.67 (0.25 - 1.81) | 0.512 |
|  |  | Weighted median | 4 | 0.93 (0.73 - 1.17) | 0.514 |
|  |  | Simple mode | 4 | 0.92 (0.67 - 1.26) | 0.636 |
|  |  | Weighted mode | 4 | 0.90 (0.65 - 1.25) | 0.586 |
| Genus | Ruminococcaceae (UCG010) | Inverse variance weighted | 4 | 0.98 (0.87 - 1.10) | 0.690 |
|  |  | MR Egger | 4 | 1.39 (0.79 - 2.45) | 0.371 |
|  |  | Weighted median | 4 | 0.97 (0.84 - 1.11) | 0.618 |
|  |  | Simple mode | 4 | 0.91 (0.75 - 1.10) | 0.401 |
|  |  | Weighted mode | 4 | 0.92 (0.77 - 1.11) | 0.451 |
| Genus | Subdoligranulum | Inverse variance weighted | 4 | 1.08 (0.97 - 1.20) | 0.168 |
|  |  | MR Egger | 4 | 0.87 (0.50 - 1.54) | 0.518 |
|  |  | Weighted median | 4 | 1.04 (0.92 - 1.17) | 0.179 |
|  |  | Simple mode | 4 | 1.02 (0.88 - 1.19) | 0.781 |
|  |  | Weighted mode | 4 | 1.03 (0.89 - 1.18) | 0.735 |

**Table S4 The heterogeneity of gut microbiota instrumental variables.**

| **Group** | **Gut microbiota** | **Cochran's Q** | **df** | **P-value** |
| --- | --- | --- | --- | --- |
| Order | Actinomycetales | 1.90 | 3 | 0.594 |
| Family | Actinomycetaceae | 1.88 | 3 | 0.597 |
| Family | Clostridiaceae 1 | 8.18 | 9 | 0.516 |
| Genus | Clostridiumsensustricto 1 | 5.22 | 6 | 0.516 |
| Genus | Haemophilus | 5.46 | 8 | 0.708 |
| Genus | Hungatella | 0.66 | 4 | 0.957 |
| Genus | Ruminococcaceae (UCG010) | 9.31 | 5 | 0.097 |
| Genus | Subdoligranulum | 9.24 | 10 | 0.510 |

**Table S5 Directional horizontal pleiotropy assessed by intercept term in MR Egger regression of the association between gut microbiota and kidney stones.**

| **Group** | **Gut microbiota** | **Egger_intercept** | **SE** | **P-value** |
| --- | --- | --- | --- | --- |
| Order | Actinomycetales | 0.01 | 0.03 | 0.815 |
| Family | Actinomycetaceae | 0.01 | 0.03 | 0.813 |
| Family | Clostridiaceae 1 | 0.01 | 0.02 | 0.732 |
| Genus | Clostridiumsensustricto 1 | -0.04 | 0.02 | 0.202 |
| Genus | Haemophilus | 0.01 | 0.02 | 0.545 |
| Genus | Hungatella | -0.04 | 0.06 | 0.531 |
| Genus | Ruminococcaceae (UCG010) | 0.01 | 0.03 | 0.686 |
| Genus | Subdoligranulum | 0.02 | 0.02 | 0.266 |

**Table S6 MR-PRESSO analysis for the association between gut microbiota and kidney stones.**

| **Group** | **Gut microbiota** | **Causal Estimate** | **SD** | **T** | **Global test P-value** |
| --- | --- | --- | --- | --- | --- |
| Order | Actinomycetales | -0.24 | 0.08 | -2.94 | 0.715 |
| Family | Actinomycetaceae | -0.24 | 0.08 | -2.95 | 0.714 |
| Family | Clostridiaceae 1 | -0.21 | 0.08 | -2.46 | 0.618 |
| Genus | Clostridiumsensustricto 1 | -0.22 | 0.09 | -2.39 | 0.460 |
| Genus | Haemophilus | 0.18 | 0.04 | 4.20 | 0.916 |
| Genus | Hungatella | -0.15 | 0.03 | -5.07 | 0.957 |
| Genus | Ruminococcaceae (UCG010) | 0.30 | 0.11 | 2.62 | 0.270 |
| Genus | Subdoligranulum | 0.22 | 0.11 | 2.13 | 0.100 |
