# Supplementary figures and images for: Causal relationship between kidney stones and gut microbiota contributes to the gut-kidney axis: a two-sample Mendelian randomization study
Source: Front Microbiol. 2023 Jul 12;14:1204311. doi: 10.3389/fmicb.2023.1204311 (PMC10368867; doi:10.3389/fmicb.2023.1204311)

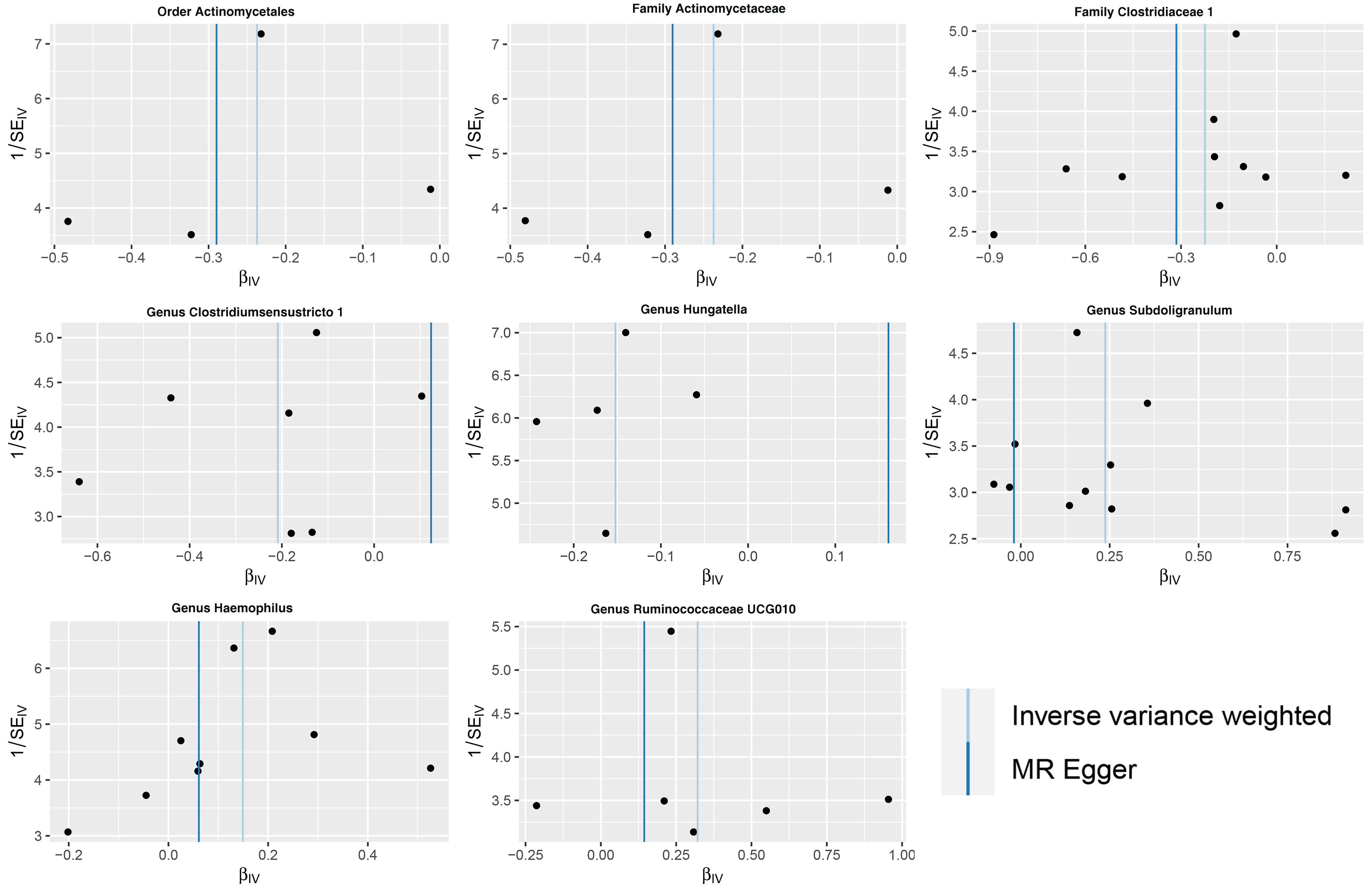

Supplement: Supplementary file 1 [file Data_Sheet_1.ZIP › Suppl.Image&Table/Figure S1.tif]

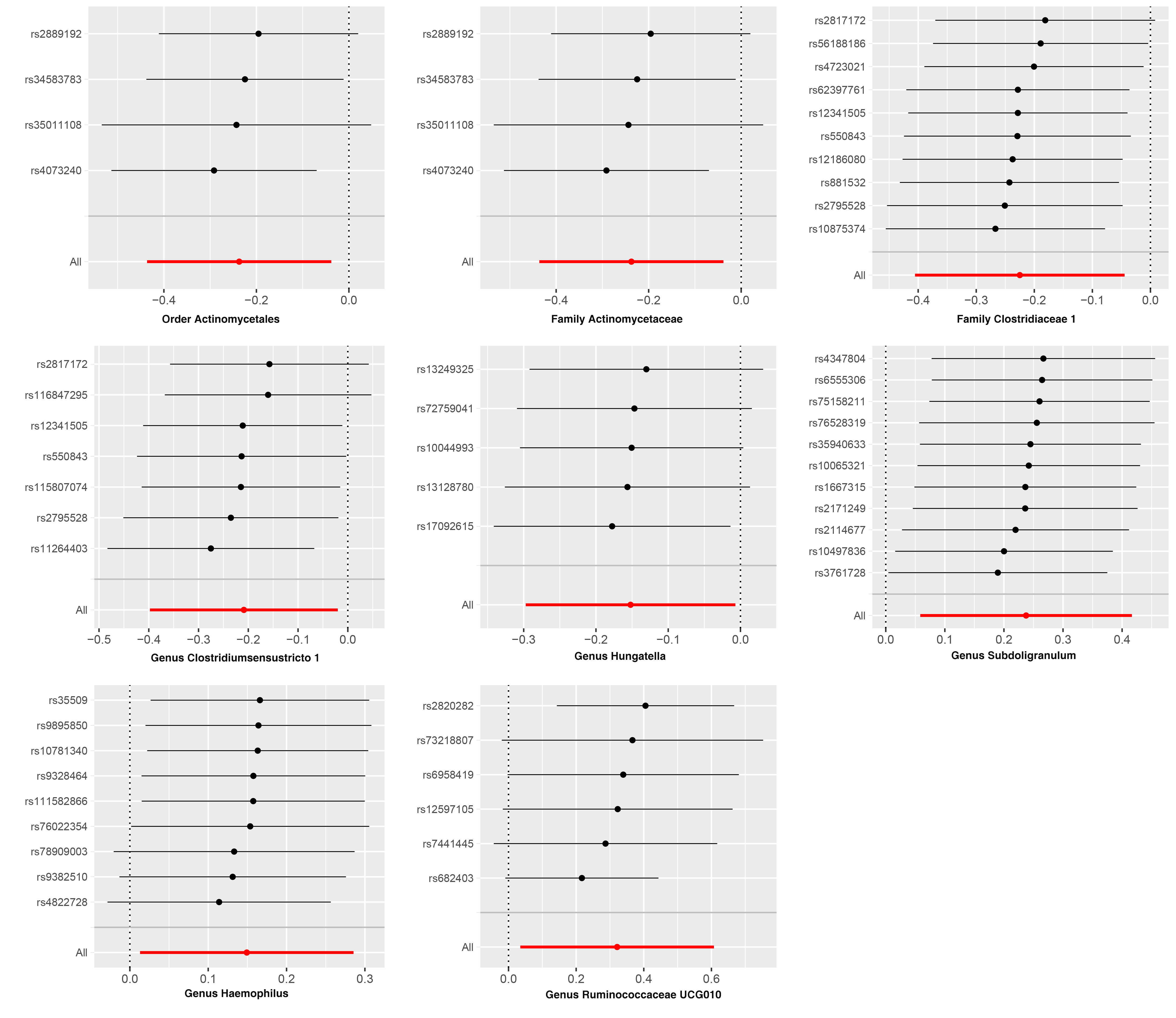

Supplement: Supplementary file 1 [file Data_Sheet_1.ZIP › Suppl.Image&Table/Figure S2.tif]

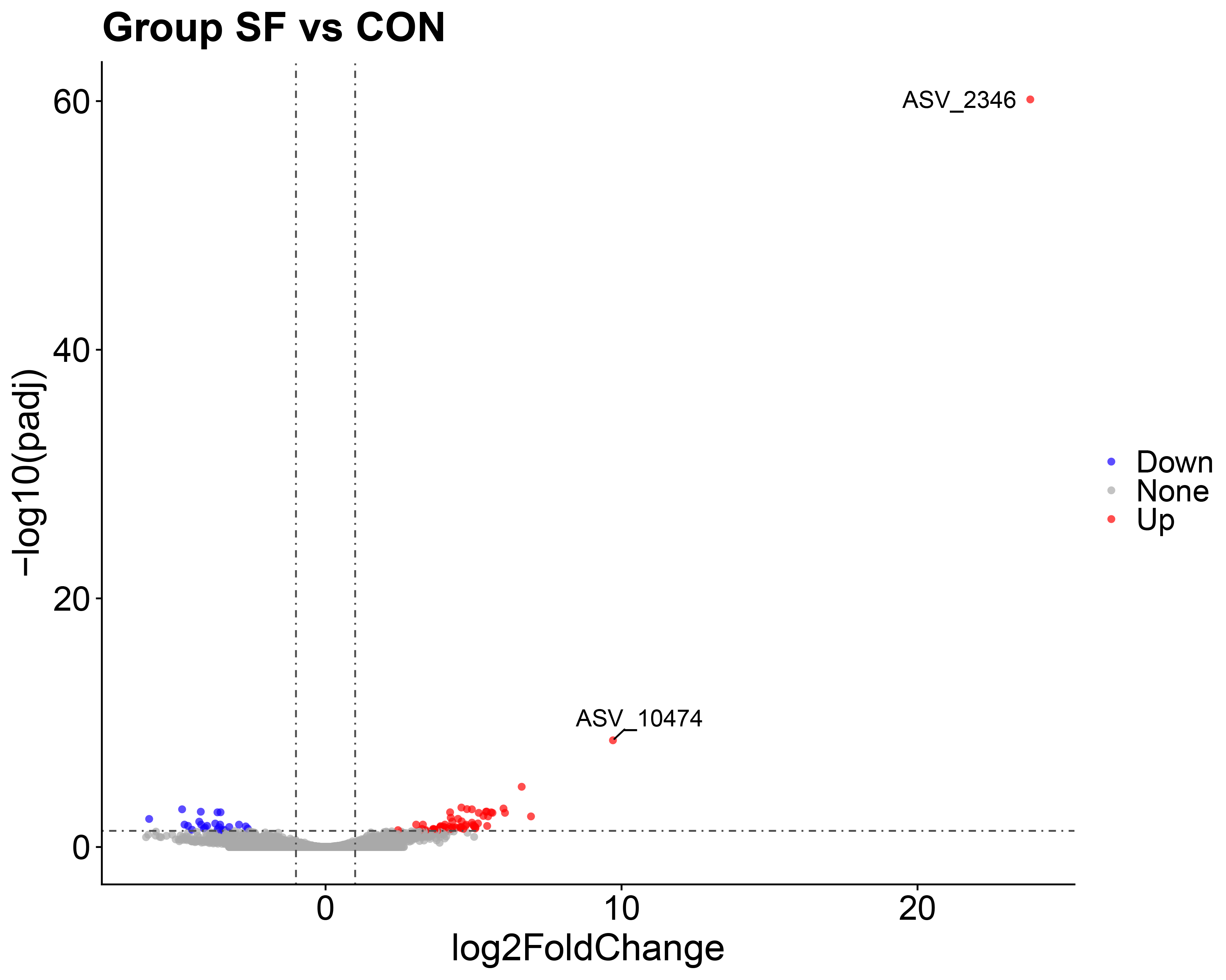

Supplement: Supplementary file 1 [file Data_Sheet_1.ZIP › Suppl.Image&Table/Figure S3.tif]
